# Home range plus: a space-time characterization of movement over real landscapes

**DOI:** 10.1186/2051-3933-1-2

**Published:** 2013-07-03

**Authors:** Andrew J Lyons, Wendy C Turner, Wayne M Getz

**Affiliations:** Department of Environmental Science Policy & Management, University of California at Berkeley, 130 Mulford Hall, Kragujevac, CA 94720-3114 USA; Centre for Ecological and Evolutionary Synthesis, Department of Biosciences, University of Oslo, P.O. Box 1066, Blindern, Oslo 0361 Norway; School of Mathematical Sciences, University of KwaZulu-Natal, Private Bag X54001, Durban, 4000 South Africa

**Keywords:** GPS, LoCoH, Movement ecology, Movement phase, Revisitation, Springbok, Time use, T-LoCoH, Utilization distribution

## Abstract

**Background:**

Advances in GPS technology have created both opportunities in ecology as well as a need for analytical tools that can deal with the growing volume of data and ancillary variables associated with each location.

**Results:**

We present T-LoCoH, a home range construction algorithm that incorporates time into the construction and aggregation of local kernels. Time is integrated with Euclidean space using an adaptive scaling of the individual's characteristic velocity, enabling the construction of utilization distributions that capture temporal partitions of space as well as contours that differentiate internal space based on movement phase and time-use metrics. We test T-LoCoH against a simulated dataset and provide illustrative examples from a GPS dataset from springbok in Namibia.

**Conclusions:**

The incorporation of time into home range construction expands the concept of utilization distributions beyond the traditional density gradient to spatial models of movement and time, opening the door to new applications in movement ecology.

**Electronic supplementary material:**

The online version of this article (doi:10.1186/2051-3933-1-2) contains supplementary material, which is available to authorized users.

## Background

Recent advances in GPS and data transmission technologies have greatly increased the volume, accuracy, affordability, and ancillary variables integrated with movement data [1, 2], creating both opportunities and challenges for ecologists [3, 4].

One of the most common uses of location data has been the estimation of home ranges and utilization distributions (UDs) [5]. Minimum convex polygons (MCPs) were among the earliest home range construction techniques, and are still widely used [6] despite their well-known biases in range estimation, sensitivity to point geometry, and inability to differentiate internal space [7–9]. In the 1980s, kernel density estimators (KDE) for constructing UDs [10] were developed and became quickly popular. These methods, based on the superposition of Gaussian or compact (e.g. uniform or Epinechnikov) kernels, are more suitable for concave geometries, can construct probability contours, and are easy to use due to their implementation in a variety of software packages [6]. More recent methods combine the simplicity of polygon methods with the robustness of kernel methods by superimposing and then aggregating non-parametric shapes constructed around each point, including Voronoi polygons [11], Delaunay triangles [12], and local MCPs [13, 14].

These classic home range methods generally treat locations as independent, an assumption especially violated with regularly sampled GPS locations. Techniques to correct for serial correlation include resampling the data [15, 16] and applying weights based on temporal density [17]. However other methods have been developed that take advantage of the information contained in serial correlation by modelling the movement between known locations. Among these are the Brownian bridge movement model (BBMM) method that constructs kernel density surfaces above each movement segment based on a diffusion model and the spatial uncertainty of each end point [18]. Enhancements to BBMM refine the bridge model between known locations by dynamically adjusting diffusion rates based on an independent segmentation of the trajectory into discrete behaviour modes [19]. Similarly, movement based KDE (MKDE) incorporates serial correlation by interpolating additional points between known locations based on activity time [20], with options to detect and correct for boundary constraints [20], and incorporate an anisotropic advective component into the local kernel [21]. More recently, time geography methods, which model movement between known locations based on the animal's maximum theoretical velocity, have been extended to home range analysis. These include the construction and aggregation of elliptical spatiotemporal potential path areas (PPA) [22], as well as probabilistic geoellipse surfaces based on a probability decay function away from the center path [23]. The later approach, known as Time Geography Density Estimation (TGDE), produces a probability surface comparable to BBMM but with smoothing objectively specified based on the animal's movement velocity.

Such movement-based home range methods explicitly incorporate information contained in temporal auto-correlation, but are still essentially models of space-use. Other methods aim to infer behavioural clues from movement data based upon the temporal patterns in the data, including variations in the amount of time spent near each location [24, 25], periodicities in step length [26, 27], path recursions [28], fractal searching behaviour [29], and a partial sum analysis of movement properties [30]. To shed light on behavioural mechanisms, such temporally-sensitive characterizations of movement can be analysed in light of data on resource distribution using spatiotemporal statistical models [31], process-based stochastic state space models [32–34], agent-based models [35, 36], and cognitive models [37].

Although progress has been made in developing methods that quantify space-use and behavior [38], these advances have not, in general, been well-integrated [39]. Home range estimators commonly ignore time other than for time-interval windowing [6, 40], while spatiotemporal and space-state models are often divorced from a model of space-use. Far fewer techniques model space-use and time-use simultaneously, with important exceptions being joint space-time utilization distributions [41] and time weighted MKDE which combines movement KDE with an adaptation of the time-of-first passage method [42].

Here we present Time Local Convex Hull (T-LoCoH) which generalizes the non-parametric utilization construction method, LoCoH [13]. T-LoCoH integrates time with space in the construction of local hulls through a scaling that relates distance and time in reference to the individual's characteristic velocity. The resulting hulls are local in both space and time, enabling metrics for movement phase and multiple dimensions of time-use including revisitation and duration. By taking hulls, rather than individual points, as samples for analysis, T-LoCoH produces UDs with high fidelity to temporal partitions of space and can differentiate internal space either with a traditional density gradient or alternately various behavioral metrics, including time-use properties. This flexibility places T-LoCoH in a growing family of methods responding to the demand for more question-based home range methods [43]. In the discussion, we compare and contrast T-LoCoH with other home range methods.

## Methods

T-LoCoH is based upon LoCoH, a non-parametric Lagrangian method for constructing UDs from a set of locations by aggregating local MCPs constructed around each point [14]. The algorithm begins by identifying a set of nearest neighbours for each point using one of three rules. The *k*-method simply selects the *k*^th^ nearest neighbours around each point. The *r-*method takes all points within a fixed radius *r*, while the adaptive *a*-method selects all points whose cumulative distance to the parent point, ordered smallest to largest, is less than or equal to *a* (Additional file 1: Figure S1). The value of *k, a* or *r* is provided by the analyst, who also decides whether duplicate locations should be ignored, deleted, or randomly offset by a fixed amount. Local convex hulls are constructed around each point and its nearest neighbours, then sorted by density which is proxied by hull area (*k*-method) or number of points enclosed with ties broken by area (*r* and *a*-methods). After sorting, hulls are cumulatively merged together by taking their union. When a union of hulls encloses *i*-percent of points, the union is saved as the *i*^th^ isopleth. The union of hulls continues until all points are enclosed, thereby providing an estimate of the 100th percent isopleth [13, 14].

### Time-scaled distance

T-LoCoH modifies the LoCoH algorithm by incorporating the time stamp of each point in two parts of the base algorithm, a) nearest neighbour selection and b) sorting of hulls.

Nearest neighbour selection is based upon a distance metric called time-scaled distance (TSD), which transforms the time interval between any two points into a third axis of Euclidean space. The translation of a unit of time into a unit of distance is accomplished through an adaptive scaling of the individual's maximum theoretical velocity, in essence a scaling of the maximum distance the individual could have theoretically traveled during the time interval. The effect of the time-distance axis is to push apart points that are far away in time even though they may be close in two-dimensional space. This transformation is not based on a mechanistic model of movement, but rather an empirical method that scales space and time in nearest neighbour identification, with space-selection at one end of the spectrum (whereby time plays no role) and time-selection at the other (space plays no role).

The equation for TSD, denoted by Ψ, with respect to any two points *i* and *j* (not necessarily in sequence) is given in Eq. 1.1

where *s* is a dimensionless scaling factor of the maximum theoretical velocity *v*_max_. All pairs of points are evaluated for nearest neighbors. When *s* = 0, the time-distance term drops out completely and TSD is equivalent to two-dimensional Euclidean distance (i.e., space selected). As *s* increases, time plays an increasingly important role, eventually reducing nearest neighbour selection to a time window. In this way TSD also bridges the continuous representation of space with discrete sampling in time.

Numerous methods exist for estimating *v*_max_, including biological studies and statistical models [22]. For the purpose of producing a heuristic yet scalable transformation of time intervals into distances, we select the simplest estimation method that is the maximum segment velocity after applying a filter to exclude temporally isolated observations.

An alternative equation for TSD, based upon a diffusive model, has also been developed and is available in the software. For the purposes of ranking nearest neighbors, the two methods are nearly identical and we focus on the simpler maximum velocity transformation in this paper. Further details on the diffusive transformation can be found in the supporting material (Additional file 1).

**Figure 1:**
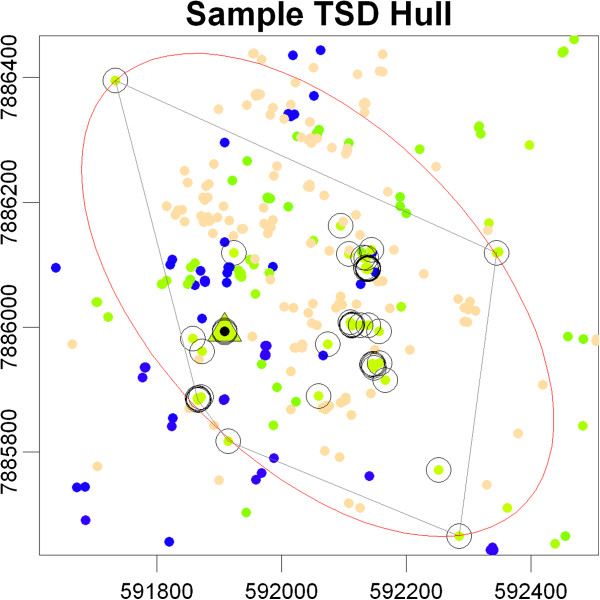
**Sample hull for a single point from a GPS dataset.** Similarly colored points represent continuity in time. The parent-point is shown by a triangle; nearest neighbours identified using TSD with *s=*0.1 are circled. Non-circled points within the hull are closer to the parent point but were bypassed as nearest neighbours due to their distance in time. The ellipse outlined in red is the bounding ellipse whose eccentricity is one of the metrics of hull elongation.

Hulls produced from neighbours identified by TSD have two properties that make them ideal units for multi-dimensional analyses of space-use. First, TSD hulls are local not only in terms of space but also time, and thus directly reflect an individual's canonical movement phase at a specific time and place [44]. These in turn correlate with geometric properties of hulls such as area and elongation. This time localization produces UDs that preserve the boundaries of spatially overlapping but temporally distinct resource patches. Second, TSD hulls often enclose points that are closer in space but are bypassed as nearest neighbours due to their distance in time (Figure 1). These enclosed points represent additional visits to the hull area, and their properties can be used to derive metrics of temporal use.

### Movement phase metrics

Because TSD-constructed hulls are local both in terms of time and space, their geometric properties may be used to help infer the animal's movement phase [44]. T-LoCoH generates two metrics of hull elongation: the perimeter-area ratio (PAR) and eccentricity of a constructed minimum volume bounding ellipsoid (Figure 1). These hull metrics do not incorporate time directly, but become meaningful measures of movement phase due to the localization of TSD hulls in space and time.

The eccentricity of an ellipse varies from 0 for a perfect circle to 1 for a line. Hulls with low PAR or eccentricity represent areas of non-directional movement, whereas a high value PAR or eccentricity indicates areas where the animal was moving directionally, such as when the animal was migrating or traversing an area with low resource value. Elongation isopleths can be constructed by sorting hulls by PAR or eccentricity, thus delineating the movement space into regions with similar elongation values.

### Time-use metrics

The amount of time an animal spends in an area, as well as the frequency of revisitation to that area, reflect two dimensions of resource value to the animal. These time-related variables can be thought of as axes of a time-use space upon which movements and resources in the landscape may be delineated (Additional file 1: Figure S2). For example, the area where an animal sleeps may have a relatively high duration (i.e., it remains there for a while when resting), but may or may not have a high revisitation index. Conversely water points may have a high revisitation index, but each visit may be of relatively short duration. Hull revisitation signatures can be used to differentiate important seasonal resources from areas of searching behavior. As illustrated in this study, time-use space also suggests an alternative approach to identifying 'core territory' which classically has been thought of spatially with definitions such as the smallest area that contains 50% of observed locations [45], deviations from a null model of uniform distribution [46, 47], or jumps in the area of isopleths [16, 48].

T-LoCoH computes metrics for revisitation and duration of use based upon an *inter-visit gap* (IVG) parameter provided by the analyst. IVG is defined as the amount of time that must pass for another occurrence within the hull to be considered a separate visit. IVG will normally be related to the periodicity of the movement behavior of interest. For example if feeding is the behavior of interest and there is a daily foraging pattern, an IVG value of 24 hours, or slightly less to account for variation in the revisit interval, would be reasonable. T-LoCoH analyzes all locations within a hull, and uses the IVG value to compute the total number of separate visits to the hull as well as the mean number of occurrences per visit. These metrics will be valid measures of revisitation and visit duration provided the IVG period is at least several times larger than the sampling frequency.

### Isopleths

To construct isopleths, local hulls are sorted by one of the hull metrics (Table 1) and cumulatively merged together. Isopleths may be defined as either quantiles of points enclosed, or as contours of values of the sort metric. Sorting hulls by point density produces traditional UDs reflecting the overall frequency of occurrence. Sorting on other metrics, such as the revisitation rate, produces spatial contours that have the same overall spatial extent but differentiate internal space by different aspects of behaviour. In addition to isopleths, behavioural patterns may emerge by exploring covariance and novel associations in the distribution of hulls in Euclidean space, hull metric space, and time.

**Table 1:** **T-LoCoH hull metrics**

***Density***	***Time use***
- Area	- Revisitation rate (number of separate visits^b^)
- Number of nearest neighbours used in hull construction	
	- Duration of visit (mean number of occurrence per visit^b^)
- Number of enclosed points	
*Elongation/movement phase*	- Revisitation rate and duration of visit normalized by area
- Eccentricity of a bounding ellipsoid constructed around the hull	*Time*
- Ratio of hull perimeter to area	- Hour of day^c^
- Mean and standard deviation of the speed^a^ of nearest neighbours used in hull construction	- Month^c^
	- Date^c^
- Mean and standard deviation of the speed^a^ of all points enclosed by the hull	- Time span of hull nearest neighbours

^a^ speed of a point sampled at time *t* is measured from *t*-1 to *t*+1.

^b^ separate visits differentiated by an inter-visit gap period provided by the analyst.

^c^ of the hull parent point.

### Simulated data

**Figure 2:**
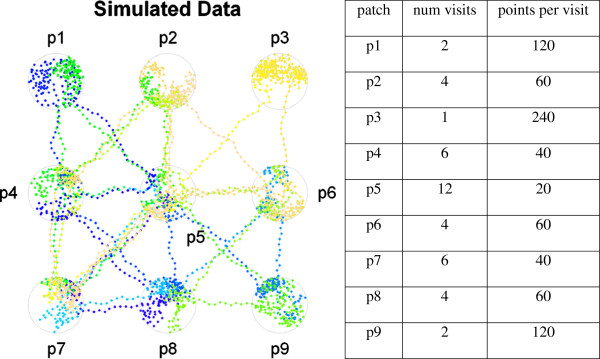
**Simulated dataset.** The simulated data represent the locations of a single individual moving among nine resource patches with a gradient of revisitation rates, durations, and directionality. Point colors represent temporal continuity.

To evaluate T-LoCoH, we constructed a simulated dataset consisting of a single animal moving with a fixed step length and sampling frequency between nine resource patches (Figure 2). Within each patch, the individual makes a pre-determined number of random steps with a constant step length and fixed sampling frequency of one hour. When it is time to move to the next patch, the animal makes directional movements to the patch exit area, also with a constant step length. It then proceeds to the next patch with a stochastic offset in the bearing applied at each step, drawn from a uniform distribution between negative and positive π/6 radians. Each patch contains roughly 240 locations but with a gradient of revisitation rates and durations.

### Springbok data

**Figure 3:**
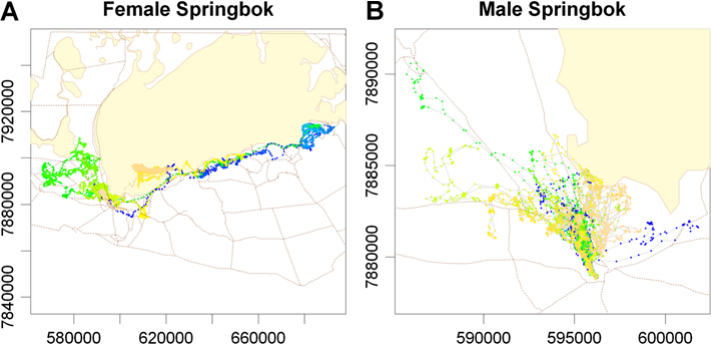
**Maps of the female (A) and male (B) springbok locations in Etosha National Park, Namibia.** The colors of the points reflect temporal contiguity; tan lines are roads; yellow polygons are salt pans.

We also applied T-LoCoH to a real dataset captured by GPS collars fitted on two springbok (*Antidorcas marsupialis*) in Etosha National Park (ENP), Namibia. Springbok are medium-sized antelope endemic to semi-arid regions of southwestern Africa. Although springbok are desert-adapted animals, able to achieve water balance through dietary sources alone, they drink water when it is available and frequently stay close to water sources during the dry season (May through October in ENP) [49, 50]. Breeding males are highly territorial while non-breeding males and females can roam significant distances [50]. Springbok in Etosha were selected as a test case for T-LoCoH due to their varied movement patterns and sharp edges in their habitat caused particularly by saltpans. Location data for one male and one female were sampled every 30 minutes beginning early September 2009 and continuing through mid-April 2010 for the male and August 2009 for the female, resulting in approximately 10,700 and 17,200 locations respectively (Figure 3, Additional file 2: Movie S1, Additional file 3: Movie S2). Location data were projected to Universal Transverse Mercator coordinates using ArcGIS [51] then imported into R.

### Implementation

We implemented T-LoCoH in the R programming language [52] because of its wide use by movement ecologists, open source license, and flexibility in connecting to spatial databases [4]. The T-LoCoH package for R includes functions to load, clean, and save datasets; identify nearest neighbours; create hulls; compute hull metrics; sort and merge hulls into isopleths; plot results; overlay vector and raster GIS data; and export outputs as graphic images, GIS layers, and animations. The T-LoCoH software requires at a minimum a set of points as input, and with this can produce all the constructions as the original LoCoH. To incorporate time into the analysis, each point also requires a time stamp.

T-LoCoH for R is best conceived of as a collection of data analysis and visualization tools rather than a one-click solution. The general workflow for using T-LoCoH is to 1) select a value of *s* that sufficiently scales the relationship between time and distance for the time scale of interest, 2) select a nearest neighbour method (*k*, *a* or *r* method) and parameter value that does the best job balancing type I and type II errors in the animal's total home range, 3) sort hulls according to density, elongation, or time use metrics depending on the questions of interest, 4) examine isopleths or hull parent points, and 5) interpret. A more detailed workflow is given in Table S1 (Additional file 1), and guidelines for parameter selection are provided in Appendix 1.

## Results

### Simulated data

Following the workflow outlined in Table S1 (Additional file 1), we first selected a span of time corresponding to a movement pattern of interest. From a priori information about how the simulated dataset was constructed, we knew the amount of time spent within a single patch visit varied from 20 to 240 hours, and we wanted to select a value of *s* such that points from separate visits to the same patch will have TSD values far enough apart to be excluded as nearest neighbours. After plotting the distribution of *s* that results in the spatial terms equaling the time-distance term in TSD (Additional file 1: Figure S3A), we selected *s*=0.3, which is close to or greater than the median value of *s* for the full range of *Δt* and results in approximately 60% of all hulls being time-selected (Additional file 1: Figure S3B).

**Figure 4:**
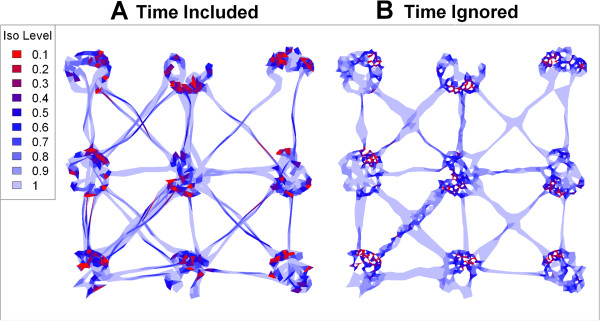
**Density isopleths for simulated data for** ***k*** **=6.** In (**A**) time is included (*s*=0.3), and (**B**) ignored (*s*=0). Isopleth levels indicate the proportion of total points enclosed. Red isopleths have a higher density of points. Note in **A** the better resolution of pathways and filling of holes.

To examine the effects of time on home range construction, we next used the *k-*method to create hulls with and without time (*s*=0 and 0.3 respectively) for a range of *k*, selected the *k* value that best satisfied the minimum spurious holes covering rule for the known patches, and constructed density isopleths. A visual comparison of isopleths reveals that the inclusion of time does a far better job delineating pathways while still capturing density gradients within the patches (Figure 4).

**Figure 5:**
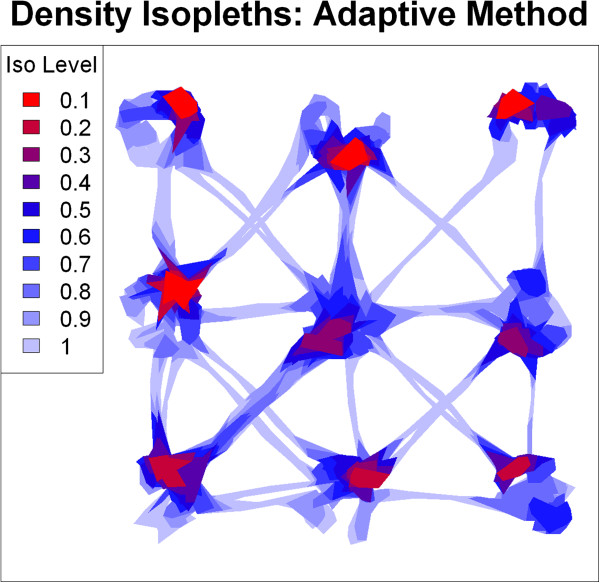
**Density isopleths for simulated data created with the adaptive method (** ***s*** **=0.3,** ***a*** **=220).** Isopleth levels indicate the proportion of total points enclosed, along a gradient of point density (red highest density, light blue lowest).

We next created hulls using the adaptive method, which does a better job minimizing spurious cross-overs caused by forays away from core areas [13]. We used the minimum proportion inclusion rule with *n*=2 and 10 to identify upper and lower bounds for *a*, created hulls for a sequence of values in this range (Additional file 4: Movie S3), and visually selected *a*=220 as the one which filled holes in core areas and minimized spurious cross-overs (Figure 5).

**Figure 6:**
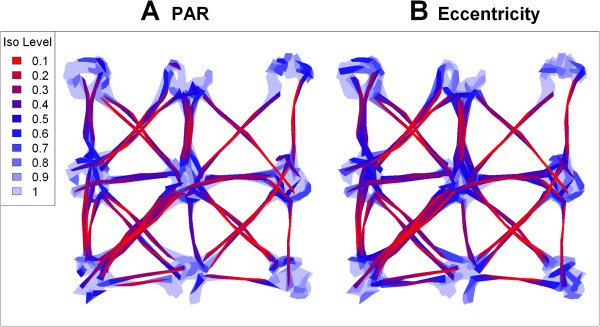
**Elongation isopleths for simulated data.** Elongation isopleths for simulated data created by sorted hulls by perimeter-area ratio (**A**) and eccentricity of bounding ellipse (**B**). Isopleth levels indicate the proportion of total points enclosed. Blue isopleths represents contours of low elongation (i.e., non-directional movement), while red indicates higher levels of elongation. Hulls constructed using the *a*-method (*s*=0.3, *a*=220).

**Figure 7:**
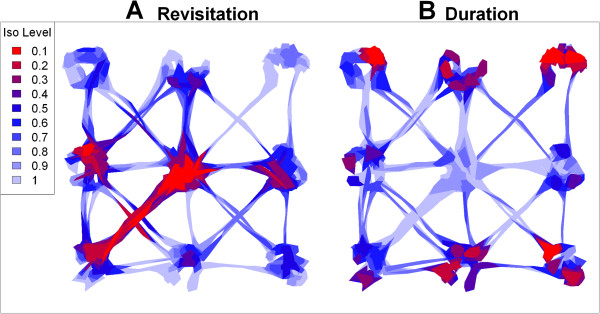
**Time-use isopleths for simulated data.** Revisitation isopleths (**A**) represent relative frequency of revisitation, with red contours being the hulls most often revisited, and light-blue the least often. Temporal duration isopleths (**B**) reflect the amount of time spent on each visit, with red indicating hulls with the longest duration and light-blue the shortest. Isopleth levels indicate the proportion of total points enclosed. Visits differentiated by an inter-visit gap period of 24 time steps, which was selected based on a priori information about the minimum period of time between patch visits. Hulls were constructed using the fixed-*a* method (*s*=0.3, *a*=220).

We then computed two hull metrics for elongation (eccentricity of the bounding ellipsoid and perimeter-area ratio) and two metrics of time-use (number of separate visits and mean number of locations per visit). For the time-use metrics, we used an inter-visit gap period of 24 time steps based on a priori knowledge of the minimum amount of time the individual would be away from a patch before another return. Isopleths created from these metrics effectively identified the gradients of directionality and time-use that were programmed into the model. Both metrics of elongation highlighted the pathways as areas of directional movement, and within patch movements as largely non-directional (Figure 6). The revisitation isopleths (Figure 7A) identified the center patch, where the individual passed through more than any other patch but for brief periods of time, as an area with a high rate of revisitation, as well as the 'highway' that was used several times to traverse between patches 5 and 7. Other areas with relatively high rates of revisitation were the 'exit area' of patches that acted as obligatory transit points between patch movements. Single-use pathways were correctly identified as the areas with the lowest rates of revisitation. Hulls with high duration values tended to be around the edges of patches where the animal was programmed to 'bounce back' off the border (Figure 7B). Hulls with the shortest duration values were along pathways and in the center transit patch.

### Springbok data

Using the same workflow as before, we began by examining the distribution of *s* that produces space-time parity for a range of time scales, as well as the proportions of time selected hulls (Additional file 1: Figure S6). Daily foraging and watering cycles are known to be strong factors in shaping space use patterns in antelope, so we selected *s*=0.01 which in both individuals is near or above the median parity value for 24 hours.

**Figure 8:**
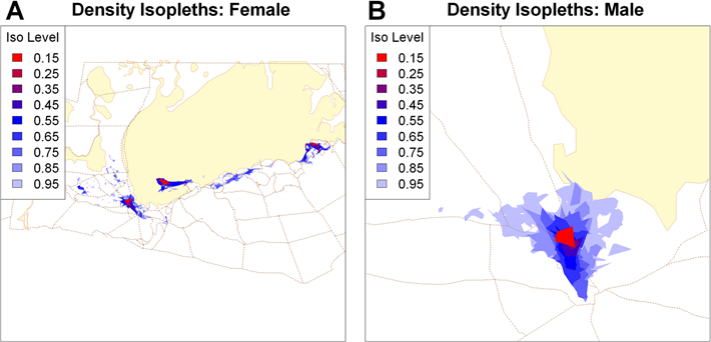
**Density isopleths for the female (A) and male (B) springbok.** Isopleth levels indicate the proportion of total points enclosed along a gradient of point density (red highest density, light blue lowest). Hulls constructed with the *a*-method (*a*=8500 and 3700 for the female and male respectively, *s*=0.01, *k*_min_=0, duplicate points offset by 1 map unit). Tan lines are roads, and yellow polygons are salt pans.

We next computed the lower and upper bounds for *a* as the minimum *a* value that include every point as a nearest neighbour in a hull with 3 and 5 points respectively, obtaining ranges 4940–9950 for the female and 2450–5100 for the male. We created hulls for a sequence of *a* values in these ranges, plotted the isopleth area and edge-area ratio curves (Additional file 1: Figure S7), and isopleth maps (Additional file 5: Movie S4, Additional file 6: S5). We made final selections of 8500 and 3700 respectively, corresponding to jumps in the isopleth area curves, local minima in the edge-area ratio curves for the lower isopleths, and a visual inspection of the isopleth maps looking for spurious hole covering and omission of real gaps (Figure 8).

**Figure 9:**
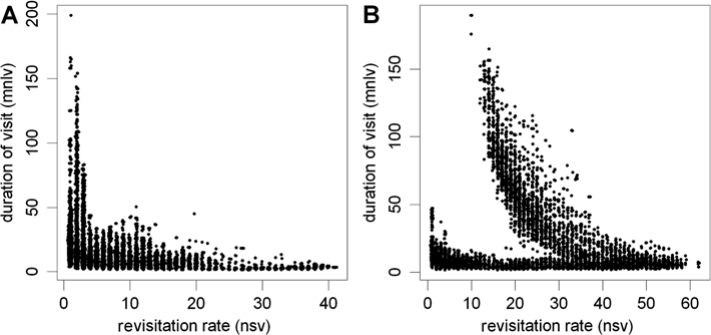
**Scatterplots of revisitation and visit duration for female (A) and male (B) springbok.** Each point represents a hull. On the *x*-axis is revisitation rate (number of separate visits). On the *y*-axis is duration of visit (mean number of locations in the hull per visit). Separate visits defined by an inter-visit gap period of 24 hours. Values have been jiggled by 0.1 to better represent point density.

**Figure 10:**
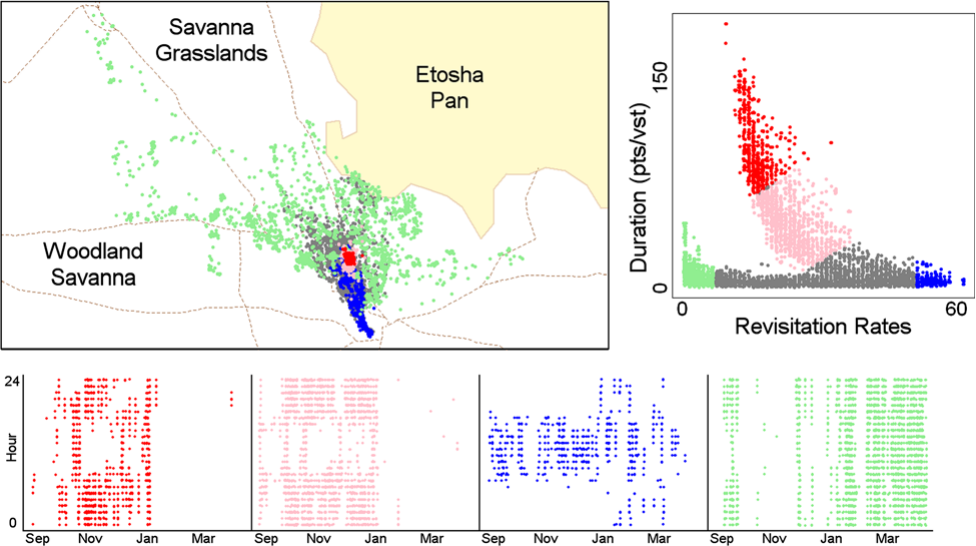
**Hull parent-points for the male springbok.** In each plot, each point represents the parent-point of a hull. The upper-right scatterplot shows the distribution of hulls by revisitation rate and visit duration. Colors from the manually defined regions of interest are reproduced on the map and bottom row of date-hour scatterplots.

Time-use metrics for the springbok were computed with an inter-visit gap period of 24 hours based on the known feeding and watering cycles of springbok. To explore the relationships among the distribution of hulls in time-use space, we produced scatterplots of the hull revisitation rates and duration (Figure 9). Striking features of these distributions include a long tail of highly revisited, low-duration hulls for the female (Figure 9A), and for the male a prominent tapering arm of hulls in the center with moderate revisitation rates and long durations (Figure 9B). To interpret these patterns, instead of creating isopleths we manually defined regions of interest in scatterplot space, then used those regions as symbology on a map of hull parent points and date-hour scatterplots (Figure 10). The results show a strong temporal signature associated with the male's territorial behavior, in which the well-defined appendage of hulls in time-use space (plot colors red and pink) coincides with a tight cluster of points on the map that radiates outward for hulls with shorter durations. The date and hour-of-day plots further reveal a diurnal pattern whereby frequently revisited hulls are used during the day for water access (blue color) with shorter movements associated with defensive behavior at night (pink color). Also evident over the course of the season are simultaneous shifts in hull durations, revisitation and the scale of movement across the landscape, indicating a shift from territorial (red/pink colors) to non-territorial behavior (green color).

**Figure 11:**
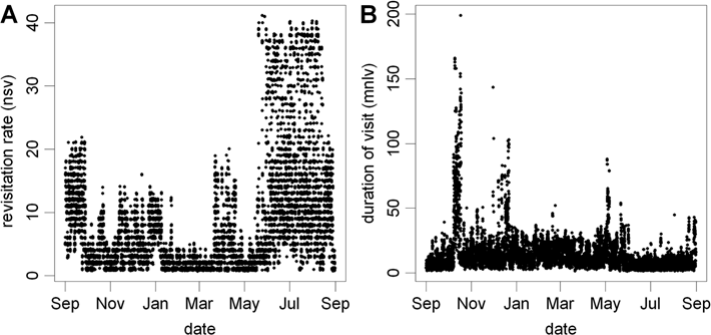
**Plots of (A) hull revisitation rate (number of separate visits), and (B) visit duration (mean number of locations in the hull per visit) over time for the female springbok.** Separate visits defined by an inter-visit gap period of 1 day. Y-values have been jiggled by 0.1 to better represent point density.

**Figure 12:**
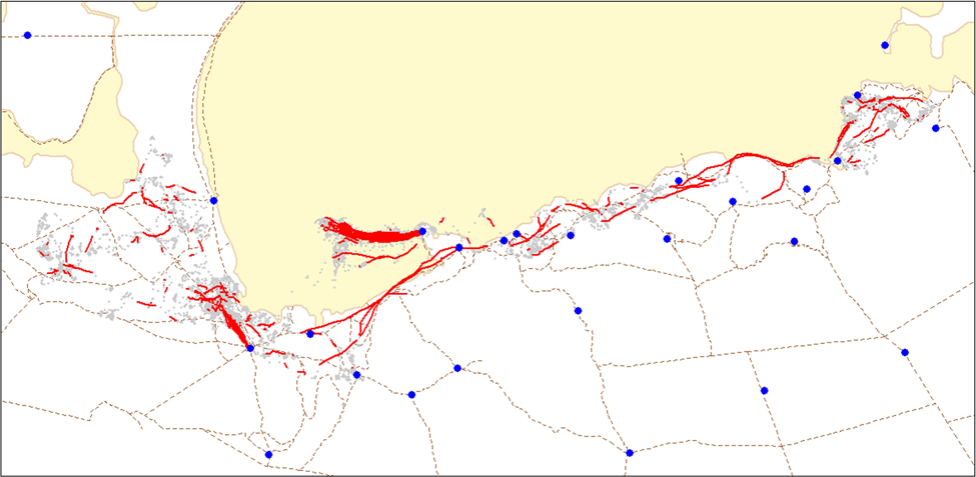
**Directional routes for the female springbok.** Routes are extracted by connecting the parent points of temporally contiguous hulls whose bounding ellipsoid eccentricity falls in the top 15%. Eccentricity values have been smoothed with a temporal averaging function and scanning window of one time step. Blue dots are known perennial water points.

Analysis of time-use metrics for the female springbok also reveals qualitatively different behaviors over the course of a year (Figure 11), reflecting adaptations to the heterogeneous distribution of resources both in space and time. These include markedly higher revisitation rates during the dry season (May-October) than wet season (November-April), indicative of seasonal dependence on perennial watering points (Figure 11A). The distribution of the average time spent per visit shows patterns of low and moderate duration interspersed with bouts of high duration, reflecting alternate periods of more stationary and migratory behavior (Figure 11B). To investigate the spatial dimensions of this alternating movement pattern, we then used hull metrics to extract 'directional routes' by connecting temporally contiguous hulls with high levels of elongation (Figure 12). These results reveal two types of directional movements, one set consisting of mostly short distances around perennial water points, and a second set of long distance movements along migratory routes.

## Discussion

Although T-LoCoH can process any set of location data, the algorithm and software implementation were developed specifically in response to the challenges and opportunities presented by GPS movement datasets. These datasets typically are large, have good temporal continuity, and follow individuals both in their core area and in inter-patch movements and excursions to new areas [5]. As a hull based method, T-LoCoH does well with GPS data due to its robustness to point geometry and spatial outliers, and ability to process relatively large datasets. Analyses of time-based hull metrics, such as revisitation rate, are sensitive to the sampling frequency and may be biased by gaps in the time series.

Our tests of T-LoCoH on a simulated dataset with known properties verified that compared to hulls created without time, density isopleths constructed from TSD hulls have better fidelity to the temporal details of movement patterns, and finer resolution of spatially overlapping but temporally differentiated resource use. This was most clearly seen around path intersections, which tend to blow up with time ignorant home range estimators but become well-defined with the TSD distance metric that penalizes points far away in time. T-LoCoH can thus produce UDs that capture not only immutable edges in the landscape such as fence lines and water bodies, but also the temporal boundaries of resource use, properties which may be advantageous when constructing space-use models for the purpose of evaluating resource utilization functions [53, 54].

Hulls that capture a comparable span of time and space also provide a basis for analysis of behavior, as demonstrated by the analysis of springbok. For the male springbok, the distribution of hulls in time-use space reveals a distinctive spike that coincides with a relatively small area we infer to be his core territory. Time-use space also reveals a diurnal pattern to movement phases, suggesting a temporal strategy for balancing resource optimization with territorial defense. In addition, hulls have the potential to serve as platforms for integrating into the analysis other fixed and dynamic variables, such as ground cover, environmental variables, proximity to landscape features, and spatial relationships with other individuals.

T-LoCoH has both similarities and differences with other home range estimation methods. Like many of the newer segment-based methods (e.g., BBMM, MKDE, TGDE), T-LoCoH incorporates the time stamp of each location rather than ignoring that information or using it to control for autocorrelation. However T-LoCoH's approach to time integration is quite different than segment-based methods, which use time information to 1) identify discrete segments of the trajectory and then 2) model movement along those segments. In contrast, T-LoCoH applies the TSD metric to characterize the spatiotemporal relationship between all pairs of points, not just sequential pairs. T-LoCoH's approach also stands apart by providing a scaling parameter that allows an analyst to control the degree to which time is involved in modeling space-use. We believe this flexibility allows T-LoCoH to be tailored to a variety of questions and systems, but additional case studies are needed to develop and test principles for space-time scaling.

Other fundamental differences between T-LoCoH and segment-based methods concern the spatial units that are aggregated and the handling of uncertainty. As a method based on hulls created by 'connecting the dots', T-LoCoH hulls by definition 'hug' the data. This produces utilization distributions that have good fidelity to edges in the movement data, including spatial edges caused by landscape features and temporal edges caused by temporal partitioning strategies. Parametric methods on the other hand do a better job at modeling spatial uncertainty, however at the cost of superimposing geometric forms that may have little to do with the actual movement patterns. Time geography methods have characteristics of both hull-based and movement-based kernel methods by modeling movement segments but with a fixed edge geoellipse defined by the maximum theoretical velocity. Another difference between polygon-based methods like T-LoCoH and parametric kernel methods is the way in which space is modeled: T-LoCoH produces vector utilization distributions whereas kernel estimators produce rasterized probability surfaces. In practice however raster surfaces can be easily converted into vector isopleths and vice-versa.

Finally, T-LoCoH differs from classic home range estimation methods in extending the concept of utilization distributions beyond that of intensity of use or probability of occurrence. Hulls, as data-driven spatial units, provide a natural foundation for a range of spatial analyses including the spatial patterns of time use strategies, activity modes, and environmental variables. Other authors have likewise begun to analyze the outputs of superimposed kernel functions for similar purposes [e.g., [42]. Time-use metrics represent the low hanging fruit of spatially explicit behavioral analyses, and we predict this trend will continue as the growing richness of geolocated ancillary data drives new research questions.

## Conclusion

For well over two decades, movement ecologists have been engaged in a lively debate about the 'best' home range estimator and efforts continue to improve the fidelity of methods with respect to the actual movements of individuals, as tested using simulation data [e.g., [55]. T-LoCoH's flexibility in generating spatial contours that reflect a variety of behavioral patterns, including but not limited to the frequency of use, departs from this search for the Holy Grail, and is rather based upon a conceptualization of home range not as a geometry to be discovered but as a biological construct inextricably linked to a question or hypothesis [5, 43]. Towards this end, we believe movement ecology will be best served by a suite of spatial analysis methods, and T-LoCoH's toolbox approach will lead to deeper insights about the underlying drivers of both space and time use.

## Appendix 1 Parameter selection

A home range is an analytical construct developed to answer ecological questions about individuals or populations, so that the best approach to parameter selection will be specific to the questions and data. T-LoCoH for R provides functions designed to help the user select and evaluate parameter values appropriate for the species, system, and study question.

The degree to which time should play a role in nearest neighbour selection depends on factors such as the degree to which temporal partitioning of resources exists, the time scale of interest, and above all the objective of the space use model. The space-time balance is controlled by the *s* parameter in the TSD equation, with two complementary approaches for selecting *s*. Viewing nearest neighbour selection as a spectrum from pure space-selection to pure time-selection, the analyst can select a value of *s* that results in a desired proportion of hulls being time-selected (Additional file 1: Figure S3A). This approach is intuitive and generally works well for producing classic home range estimates with strong fidelity to temporal partitioning. Alternately, if there is a specific time scale of interest, the analyst can plot the distribution of *s* values that equalizes the spatial and time-distance terms in TSD for all pairs of points *Δt* apart (Additional file 1: Figure S3B), in other words the values of *s* given by (cf. Eq. 1):2

With the distribution of space-time parity as a guide, the user can select a value of *s* such that time either dominates TSD for the time scale of interest, or is more balanced with distance. Other plots that aid in the selection of *s* include the ratio of time-distance to TSD or Euclidean distance (Additional file 1: Figure S4), and the time span of nearest neighbours for different values of *s* (Additional file 1: Figure S5). These distributions show how time comes to dominate space in hull construction with increasing values of *s*.

After *s* is selected, the analyst must next pick a nearest neighbour selection method. The *k*-method is intuitive and works well when there is good temporal coverage, however the adaptive or *a*-method, in which all locations within a cumulative distance *a* are considered nearest neighbours, has been shown to be the most robust to point geometry and is generally recommended [13]. The fixed radius *r*-method is appropriate for specific questions such as models of sensory space, but generally performs poorly for utilization distributions. Selecting a value for *a* or *r* is not intuitive when time is included because TSD is no longer a physical distance, so a heuristic approach is taken using visualization and computational aids. Whichever method is used, four key principles and a set of computations and visualizations guide the choice of parameter values.

The minimum proportion inclusion (MPI) rule specifies a lower limit for *a*/*k/r* as the value that results in a proportion *p* of points included as a nearest neighbour for at least one hull with *n* nearest neighbours, where *p* and *n* are provided by the analyst. If the study question calls for a space-use model for all observations, *p* would normally be 1, however if there are spatial outliers in the data or the study question concerns core areas only, *p* may be less than one. For the *k*-method, the MPI rule is satisfied by a lower bound of *k*=*n*, while the lower bound for the *a*-method is computed from the data. The MPI rule can also be used to identify an upper bound by setting *n*≥10 because *k* values in this range typically begin to over-estimate home ranges.

The minimum spurious hole covering (MSHC) rule states that the parameter value should be the smallest value that covers spurious holes, thus tending to reduce Type I errors [14]. Spurious holes are holes created by small parameter values that produce a Swiss-cheese pattern (Figure 5B), as opposed to real holes created by topography or landscape features that the animal avoided. Good places to identify spurious holes are core areas (isopleth levels ≤ 0.5) with homogenous land cover. Conversely the true hole exclusion principle provides a criterion for the upper limit by omitting areas not used by the animal hence tending to reduce Type II errors. As *a* and *k* increase, isopleths typically intrude into areas precluded by landscape boundaries such as topography or water edges, or may erroneously append large swaths of habitat in areas where the animal only traversed. Such crossover errors are usually evident as sharp jumps in plots of isopleth area (Additional file 1: Figure S7) and visual inspection of isopleth maps (Additional file 4: Movie S3, Additional file 5: Movie S4, Additional file 6: Movie S5) in reference to knowledge of the species and ecosystem.

## Electronic supplementary material

Additional file 1: **Additional figures and tables.** (DOC 458 KB)

Additional file 2: **Animation of the female springbok's movements.** Tan lines are roads, yellow polygons are salt pans, and blue dots are known perennial water points. (MOV 3 MB)

Additional file 3: **Animation of the male springbok's movements.** Tan lines are roads, yellow polygons are salt pans, and blue dots are known perennial water points. (MOV 4 MB)

Additional file 4: **Utilization distributions for the simulated data for values of** ***a*** **between 20 and 250,** ***s*** **=0.3.** (MOV 636 KB)

Additional file 5: **Utilization distributions for the female springbok for values of** ***a*** **between 4000 and 11000,** ***s*** **=0.01.** Tan lines are roads and yellow polygons are salt pans. (MOV 139 KB)

Additional file 6: **Utilization distributions for the male springbok for values of** ***a*** **between 2100 and 4500,** ***s*** **=0.01.** Tan lines are roads and yellow polygons are salt pans. (MOV 180 KB)
