# Supplementary material for: Home range plus: a space-time characterization of movement over real landscapes
Source: Mov Ecol. 2013 Jul 3;1(1):2. doi: 10.1186/2051-3933-1-2 (PMC4337754; doi:10.1186/2051-3933-1-2)
Supplement: Supplementary file 1 — Additional file 1: Additional figures and tables. (DOC 458 KB) [file 40462_2012_2_MOESM1_ESM.doc]

Additional Material for:

**Home range plus: A space-time characterization of movement over real landscapes**

Andrew J Lyons, Wendy C Turner, & Wayne M Getz

Additional material for this manuscript includes the following:

Additional Text

Table S1

Figures S1-S7

Alternative Time-Scaled Distance: The Diffusive Model

Time-Scaled Distance (TSD) is a metric that measures closeness between any two points in terms of their relationship in both space and in time. The metric begins with Euclidean distance and adds a term that transforms the temporal distance into units of space, such that points further apart in time have a larger TSD value. In T-LoCoH, TSD is used to rank each point's nearest neighbors for the purpose of creating hulls that are local in both space and time. TSD also uses a scaling parameter to control the balance between time and distance, because the degree to which time localization is helpful varies with the goals the analysis.

We explored two variants of TSD, a maximum velocity transformation presented in the main text and a diffusion-based transformation presented below. For the purposes of ranking nearest neighbors and scalability, both variants were very similar, so we recommend the simpler maximum velocity transformation. In this section, we present the derivation of the alternative diffusion model and discuss its properties. Both variants of the TSD metric are available in the T-LoCoH package for R.

The diffusive transformation is based upon a Gaussian diffusion model that computes the expected distance the animal would have travelled during the time interval had it been moving in an unbounded random walk. It can be shown that in a purely unbounded random walk with constant step length *λ* and no correlation between the direction of successive steps that the expected value of the square of the net displacement *D* between any two points *N* steps apart is given by equation 1 (Bovet & Benhamou 1988:428).

$E(D^{2})=\lambda^{2}N$ Eq. S1

Modifying this equation for a series of time-stamped locations, we estimate the expected mean square displacement of any two points separated by time interval Δ*t* by:

$E(D^{2})=\bar{d}^{2}\frac{\Delta t}{\tau}$ Eq. S2

where *τ* is the median sampling interval (i.e., dominant sampling frequency) of the dataset, and is the median step length of the entire dataset. We expect this approximation to hold well for time series in which the sampling frequency is fairly regular. Incorporating diffusion distance as a third axis in Euclidean space, and adding a dimensionless scaling factor *s* that controls the degree to which time scales space, we arrive at the diffusive form of TSD, denoted by Ψ, with respect to any two points *i* and *j* (not necessarily sequential):

Eq. S3

When *s* = 0, the diffusion term drops out completely and TSD is equivalent to two-dimensional Euclidean distance. As *s* increases, time plays an increasingly important role. For *s*>> 1, the importance of space disappears and the method reduces to a type of time‑series analysis.

Tests with simulated and real data (not presented) reveal few differences in the ranking of nearest neighbors identified with the maximum-velocity and diffusive time-space transformations, when the scaling parameter is selected to achieve a common percentage of time-selected hulls. The diffusive transformation may be better suited for analyses over longer time scales when diffusion is a more suitable model, or for species more likely to be governed by diffusion (e.g. dispersing organisms).

The diffusion transformation also provide the ability to plot predicted vs. actual distance, which while ancillary to hulls-based analyses provide useful characterizations of movement in terms of sub-diffusion and supra-diffusion. Figure S8 shows the predicted vs. actual distances between pairs of points for the two springbok tracking datasets. Displacement of the female, who travelled nearly 120km east during the wet season, exhibited a supra‑diffusion pattern with numerous plateaus and jumps reflecting long periods of foraging interspersed with short bursts of travel (Movie S1 Additional file 2). In contrast the male springbok displayed a sub-diffusion pattern for all time intervals, reflecting his markedly territorial behavior (Movie S2 Additional file 3).

Table S1

Twelve-step workflow for the T-LoCoH R package

| 1. Import data. 2. Inspect the distribution of locations, sampling frequencies and step lengths, taking note of short-timed bursts, temporal gaps, and spatial outliers. Replace bursts with single points if needed. 3. In reference to the study question, select a value of *s* that balances time and space using one of two approaches:    1. Plot the distribution of *s* vs. the proportion of time-selected hulls, then select the *s* value corresponding to the desired proportion of time-selected hulls (for UDs try 40-60% time-selected hulls)    2. Based on *a priori* knowledge of the organism, identify the time interval that corresponds to a movement pattern or cycle of interest (e.g., watering or foraging). If *a priori* knowledge does not exist, plot point distance to centroid over time to look for natural frequencies. Examine the distribution of *s* values that balance the spatial terms and time term in TSD (Equation 2) then select a value of *s* around the median value for the time interval(s) of interest. 4. Pick a nearest-neighbor selection method:    1. a-method: recommended in most cases due to robustness to point geometry    2. k-method: more intuitive and faster but may be sensitive to spatial outliers    3. r-method: may be appropriate for studies of perception, but generally not recommended for space-use models 5. Create hulls for a range of *a* or *k* values:    1. a-method: lower bound should be equal to or less than the value of *a* for which every point is a nearest neighbor in a hull composed from at least three points; and 5-10 points for the upper bound    2. k-method: start with *k* values 3, 6, 9, …24 6. Sort hulls based on number of enclosed points (a-method) or area (k-method) and progressively take the union to form isopleths. 7. View plots of:    1. isopleth area vs. *a* or *k*, looking for jumps in area that may indicate a spurious crossover    2. isopleth edge:area ratio vs. *a* or *k*, looking for minima in core areas (isopleth levels ≤ 0.5)    3. isopleth maps 8. Select by eye the value of *a* or *k* that a) minimizes spurious holes covering, b) maximizes hole filling in the core areas, and c) concurs with knowledge of the landscape / species. 9. Compute any additional hull metrics needed for the ecological question of interest. If time use metrics are needed, select a value for the inter-visit gap based on *a priori* knowledge or cycles observed in a plot of point-to-centroid distance over time. 10. Sort hulls according to the metric of interest and create isopleths. Plot isopleths overlaid with GIS layers / images as needed. 11. Create scatterplots of hull metrics, looking for novel associations. Manually digitize regions of interest or use the color wheel symbology to visualize their distribution on a map. 12. Interpret or explore associations with environmental and other data. |
| --- |

Figure S1

Illustration of methods for determining how many nearest neighbors to select around each parent point. In each panel, the center point at the center is the parent point. The *k*-method selects the nearest *k* points (**A**). The *r*-method selects all points within a fixed radius *r* (**B**). The adaptive method selects all points whose cumulative distance, ordered smallest to largest, is ≤ *a* (**C**). *k*, *r* and *a* are parameters provided by the analyst.

Figure S2

Time-use space defined by revisitation and duration axes.


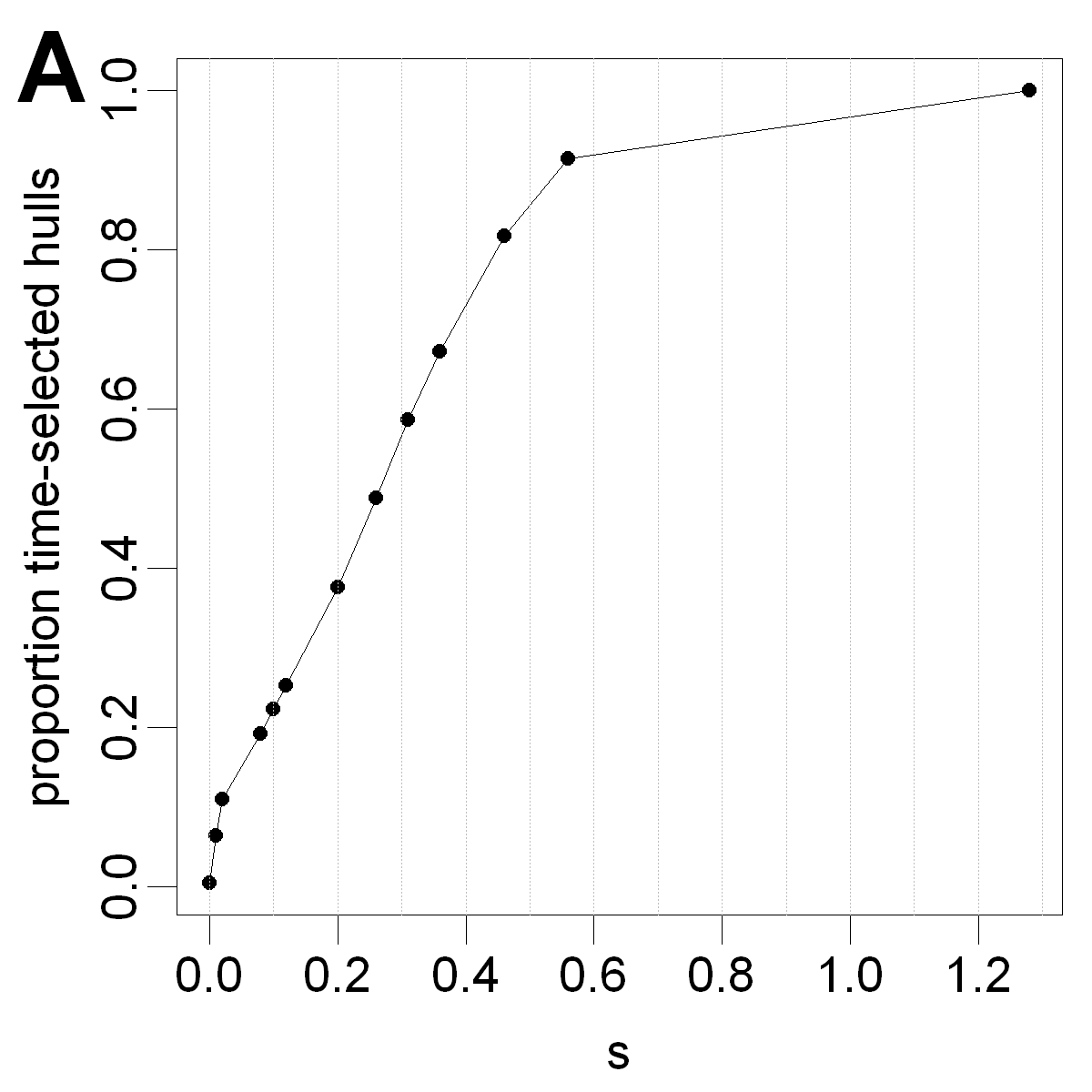

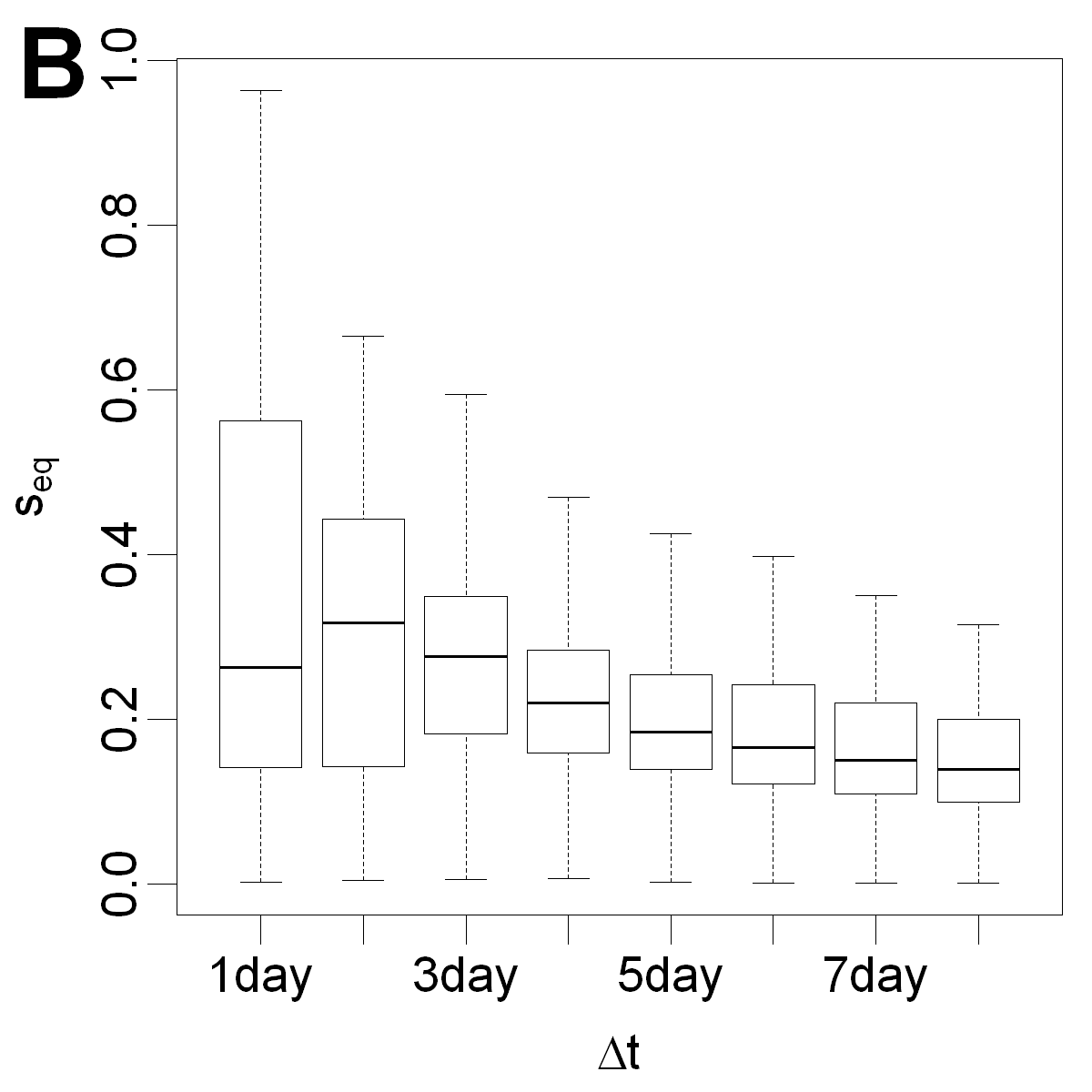


Figure S3

The proportion of time-selected hulls (i.e., hulls constructed from time sequential locations) for a range of *s* values when k=10 (**A**). Box-and-whiskers plot from the simulated dataset of the values of *s* that satisfy the equation $\left(sv_{\max}\Delta t_{ij}\right)^{2}=\Delta x_{ij}^{2}+\Delta y_{ij}^{2}$ for all pairs of points Δ*t* apart (**B**). The median value at each time interval Δ*t* represents the value of *s* whereby the time-distance term in TSD (Eq. 1) is equivalent to the actual Euclidean distance.


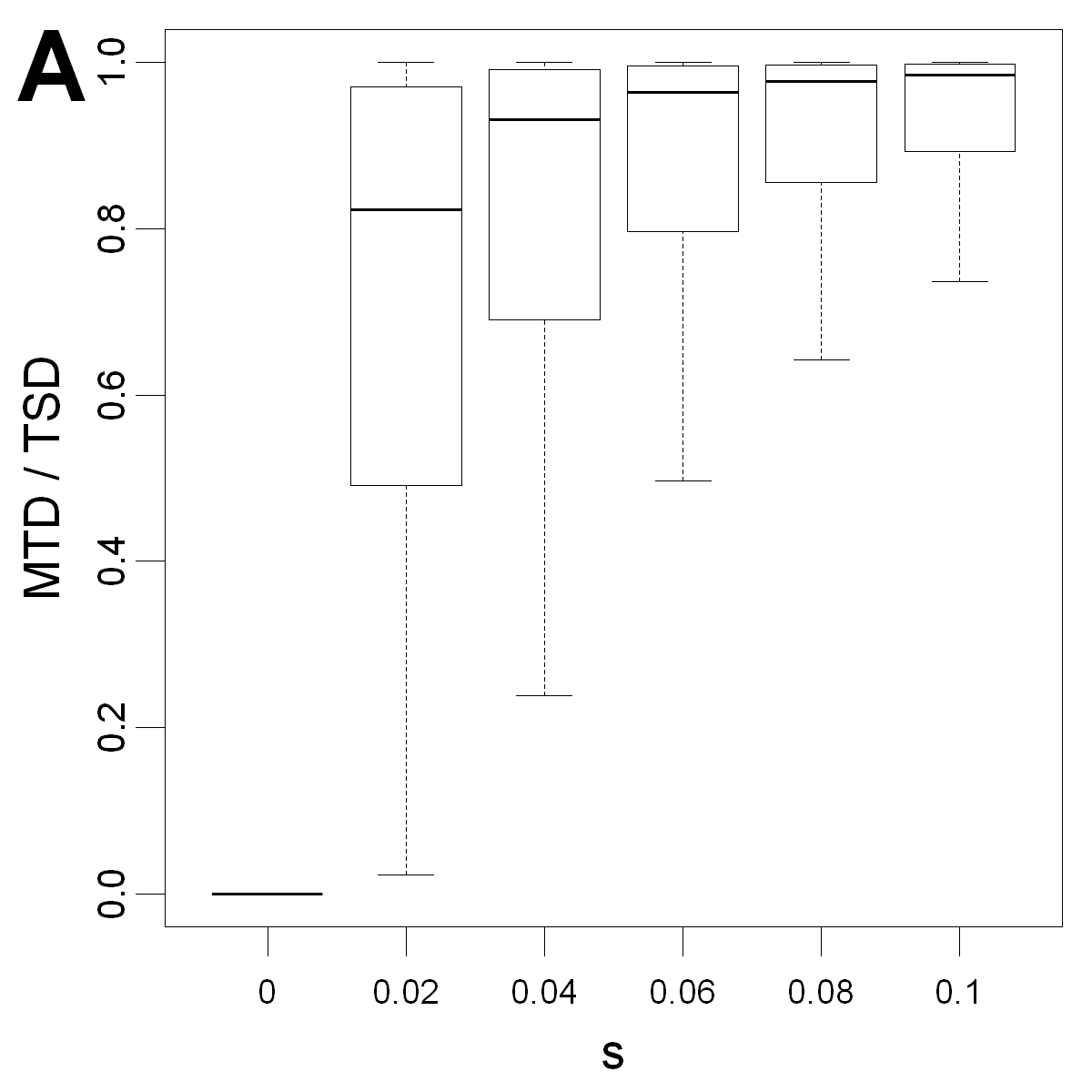

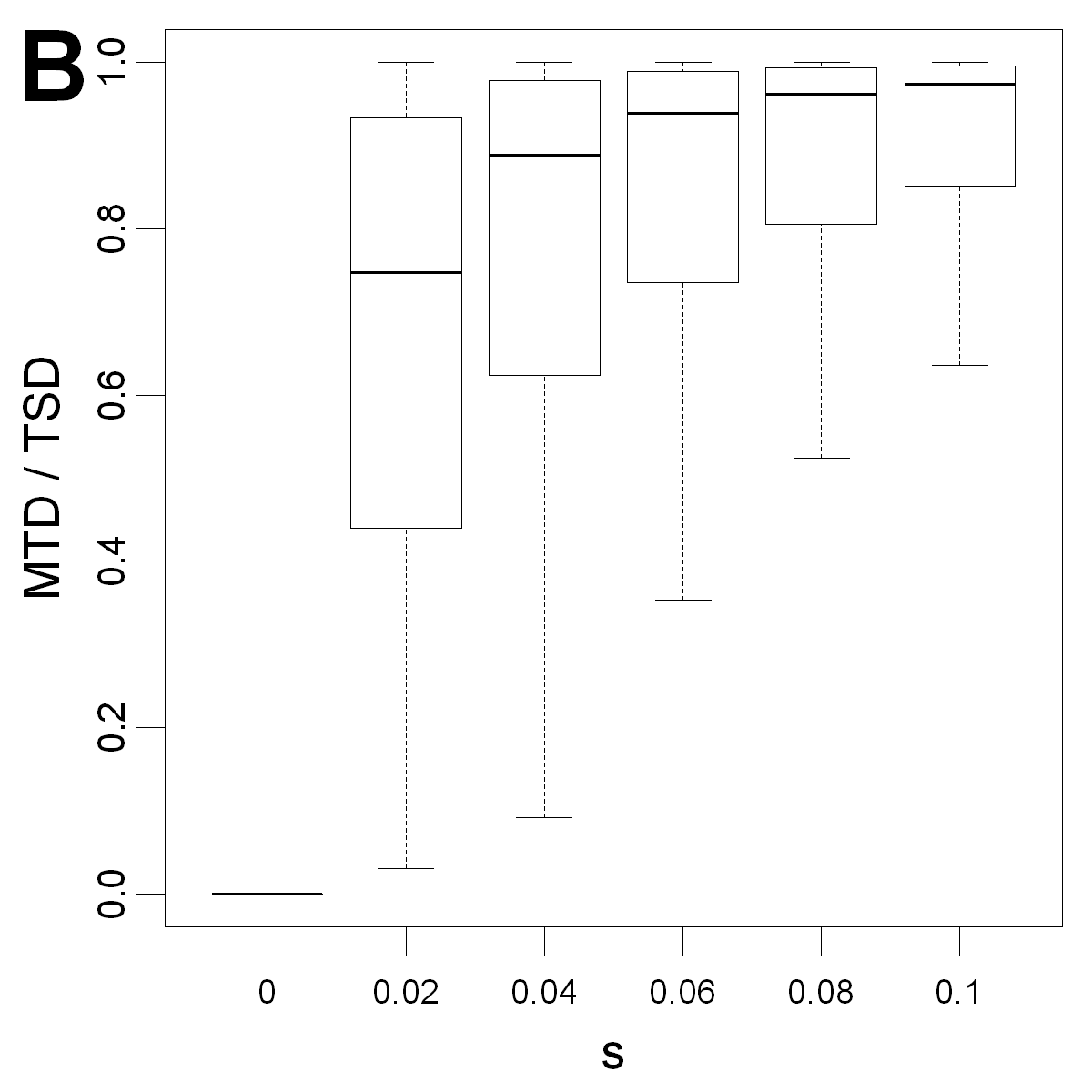


Figure S4

Box-and-whiskers plots of the ratio of the maximum temporal distance (MTD) to TSD for all nearest neighbors for *k*=10 and a range of *s* values for the female (**A**) and male (**B**) springbok. The plots reveal that as *s* increases, the time-distance term comes to dominate the TSD metric resulting in hulls which are increasingly more localized in time than space.


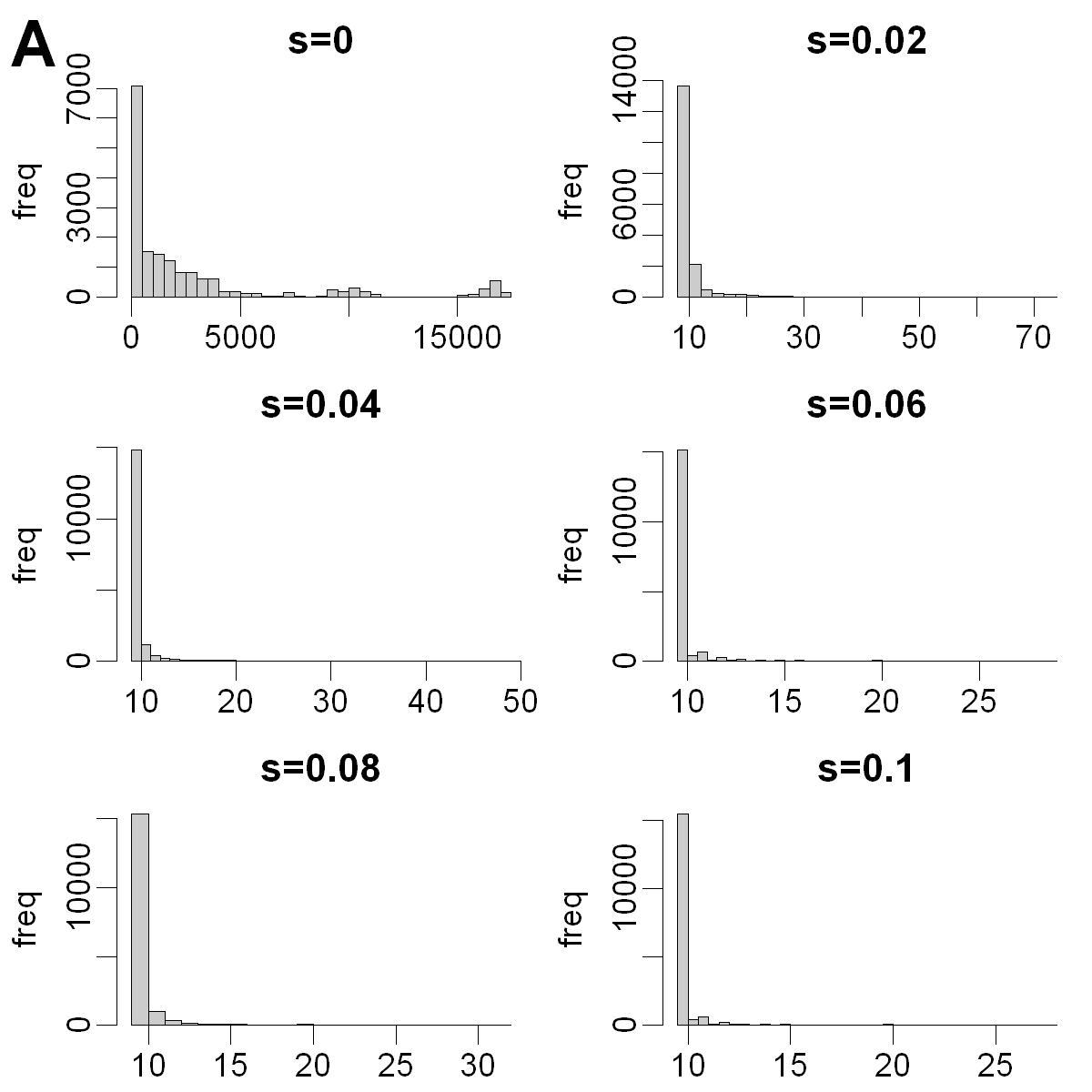

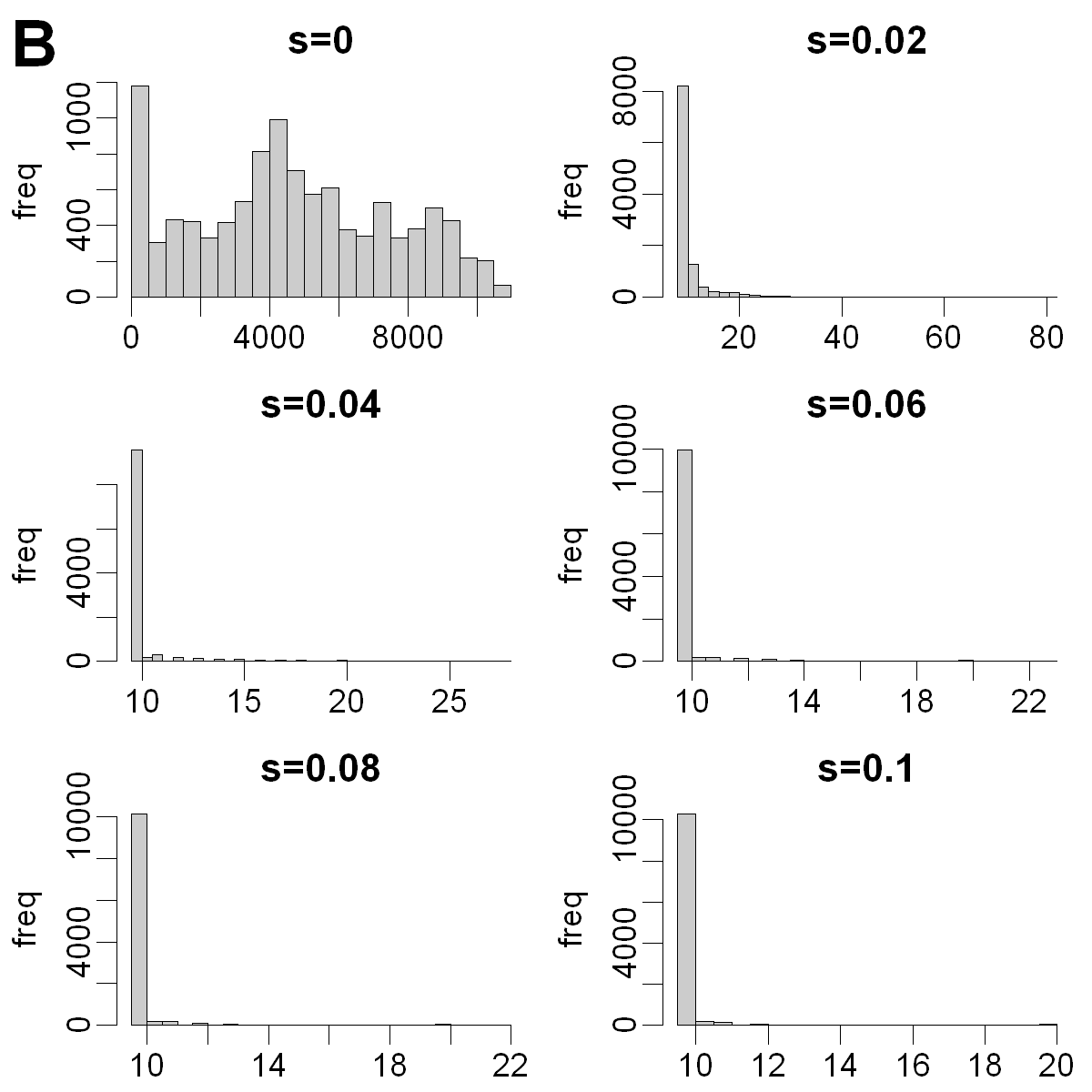


Figure S5

Histograms of the time span (in hours) of the ten nearest neighbors for each location of the female (**A**) and male (**B**) springbok (*N*=17206 and 10702 respectively), expressed as a proportion of the median sampling interval. When *s* = 0, time plays no role in the selection of nearest neighbors and the time span of a set of neighbors can even equal the duration of the entire dataset (i.e. the first and last locations can be nearest neighbors if they're close together in space). As *s* increases, the time span converges on the number of nearest neighbors in the hull, in this case *k*+1.

**
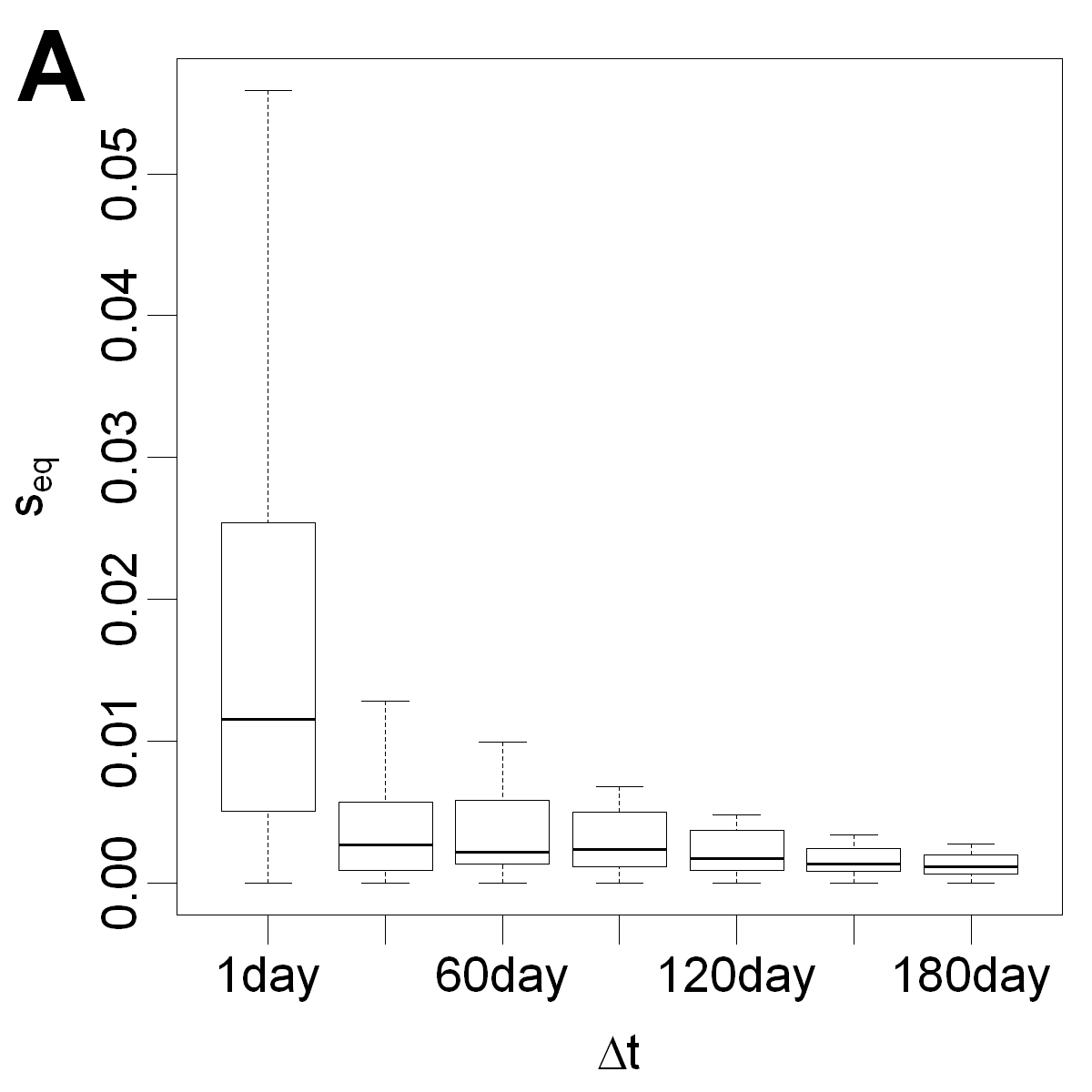

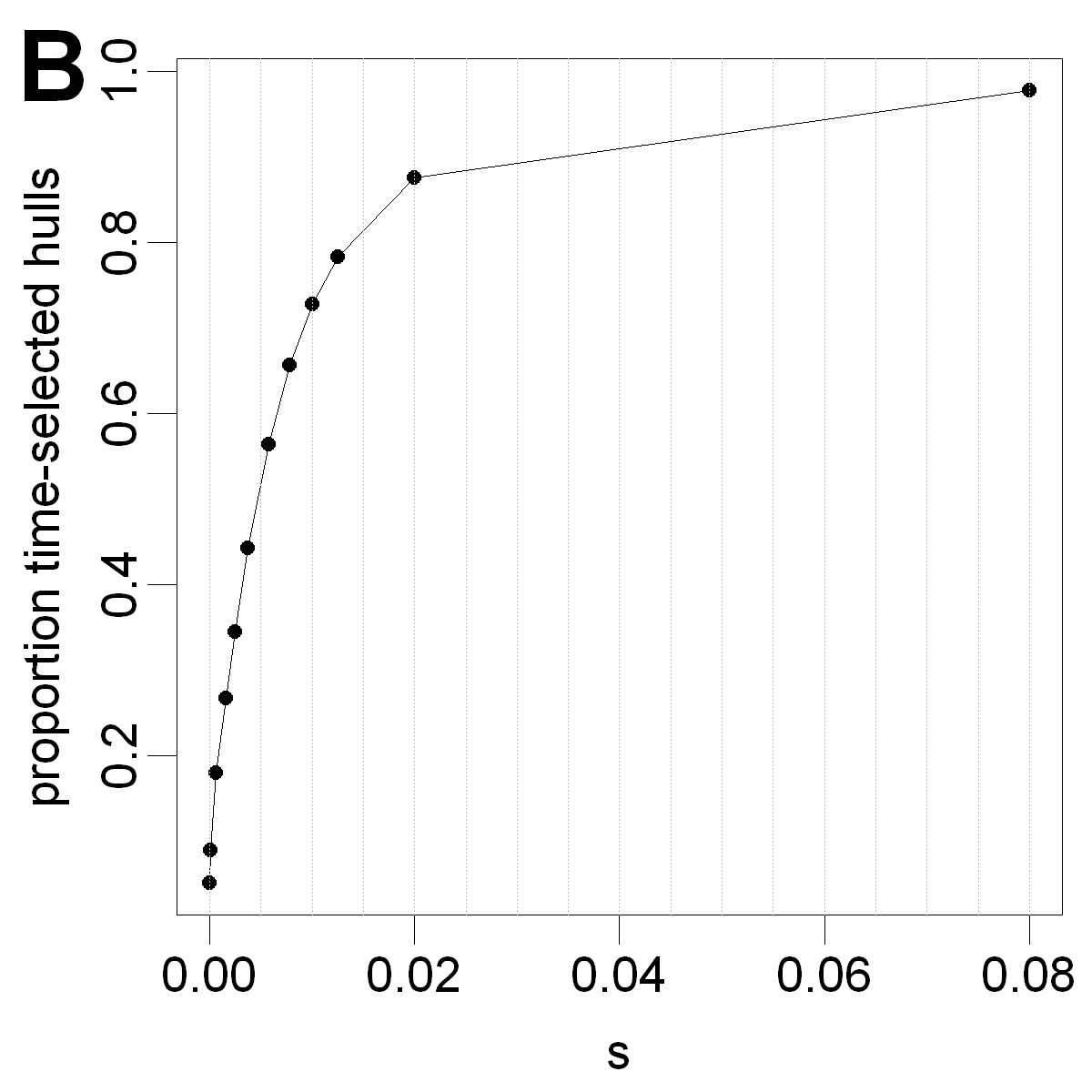
**

**
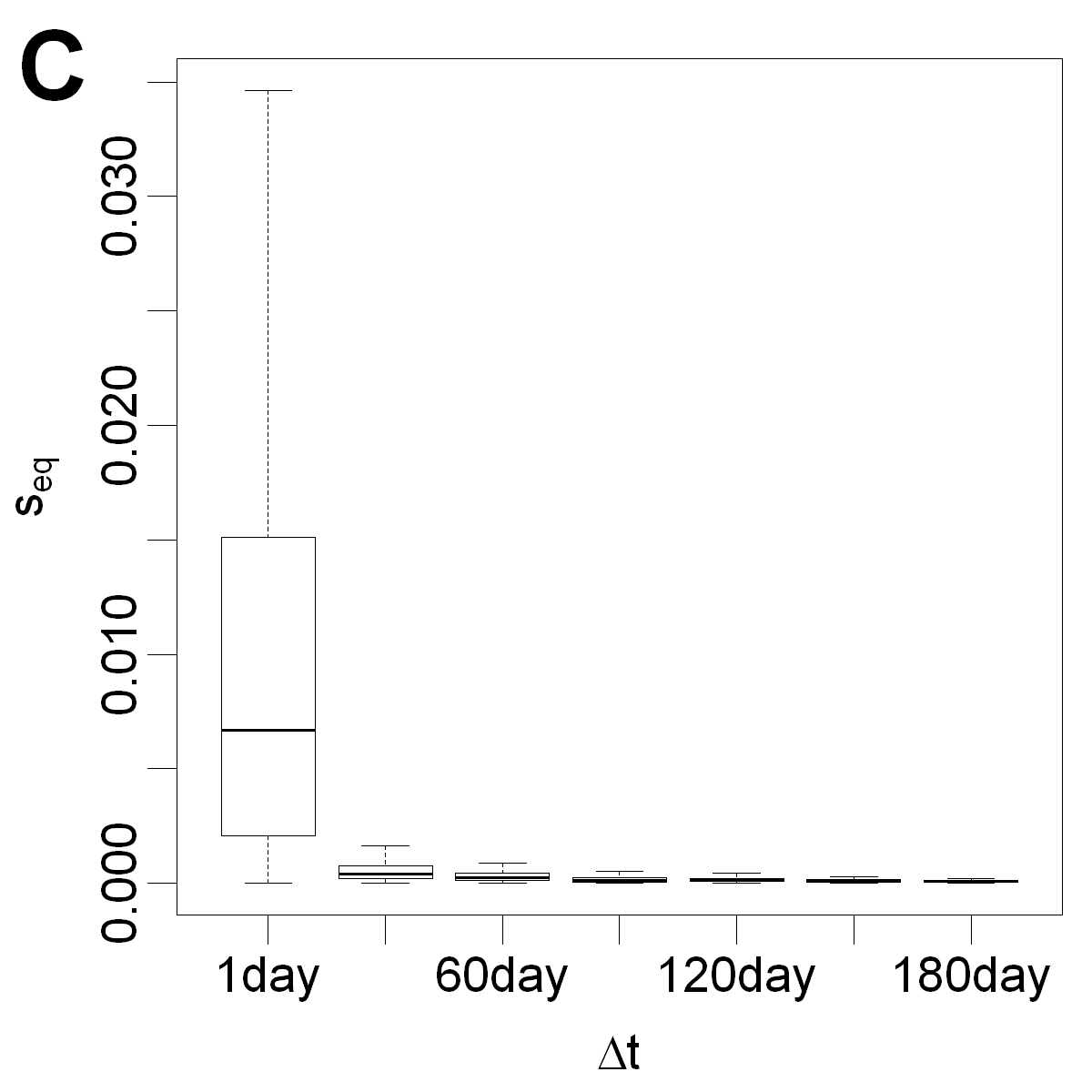

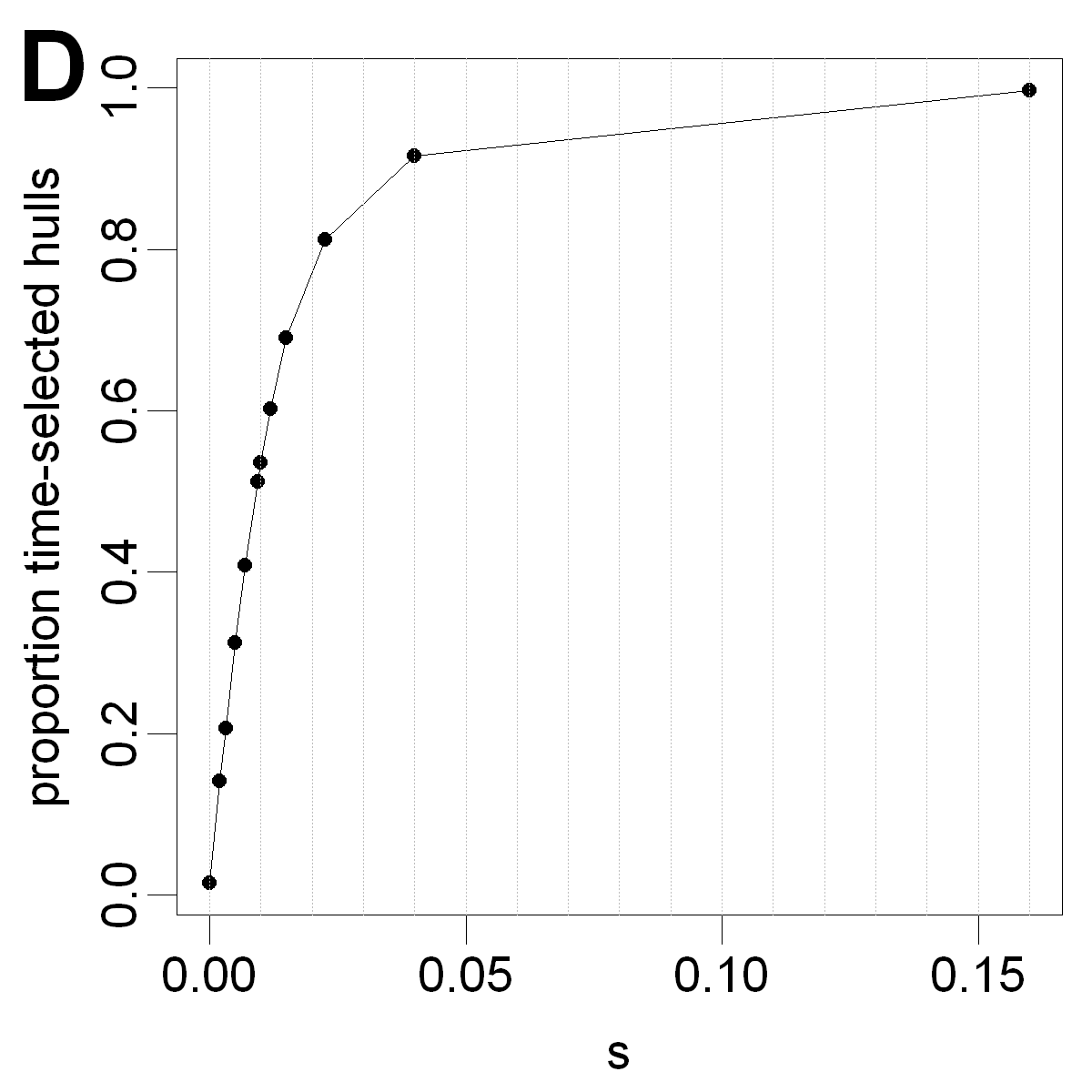
**

Figure S6

Left: box-and-whiskers plots of the values of *s* that satisfy the equation $\left(sv_{\max}\Delta t_{ij}\right)^{2}=\Delta x_{ij}^{2}+\Delta y_{ij}^{2}$ for all pairs of points Δ*t* apart for the female (**A**) and male (**C**) springbok. Right: the proportion of time‑selected hulls (i.e., hulls constructed from time sequential locations) for a range of *s* values when *k*=10 for the female (**B**) and male (**D**) springbok.


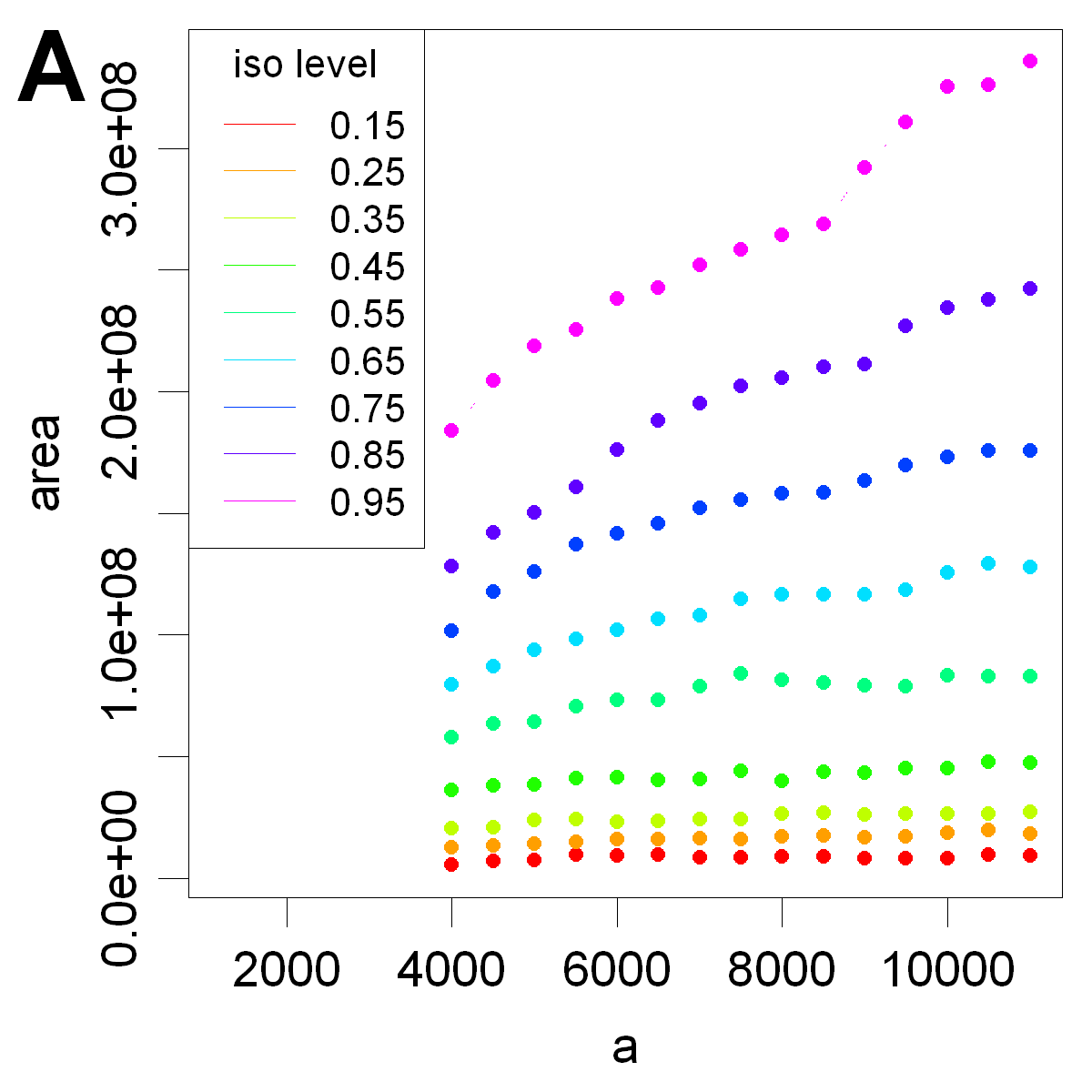

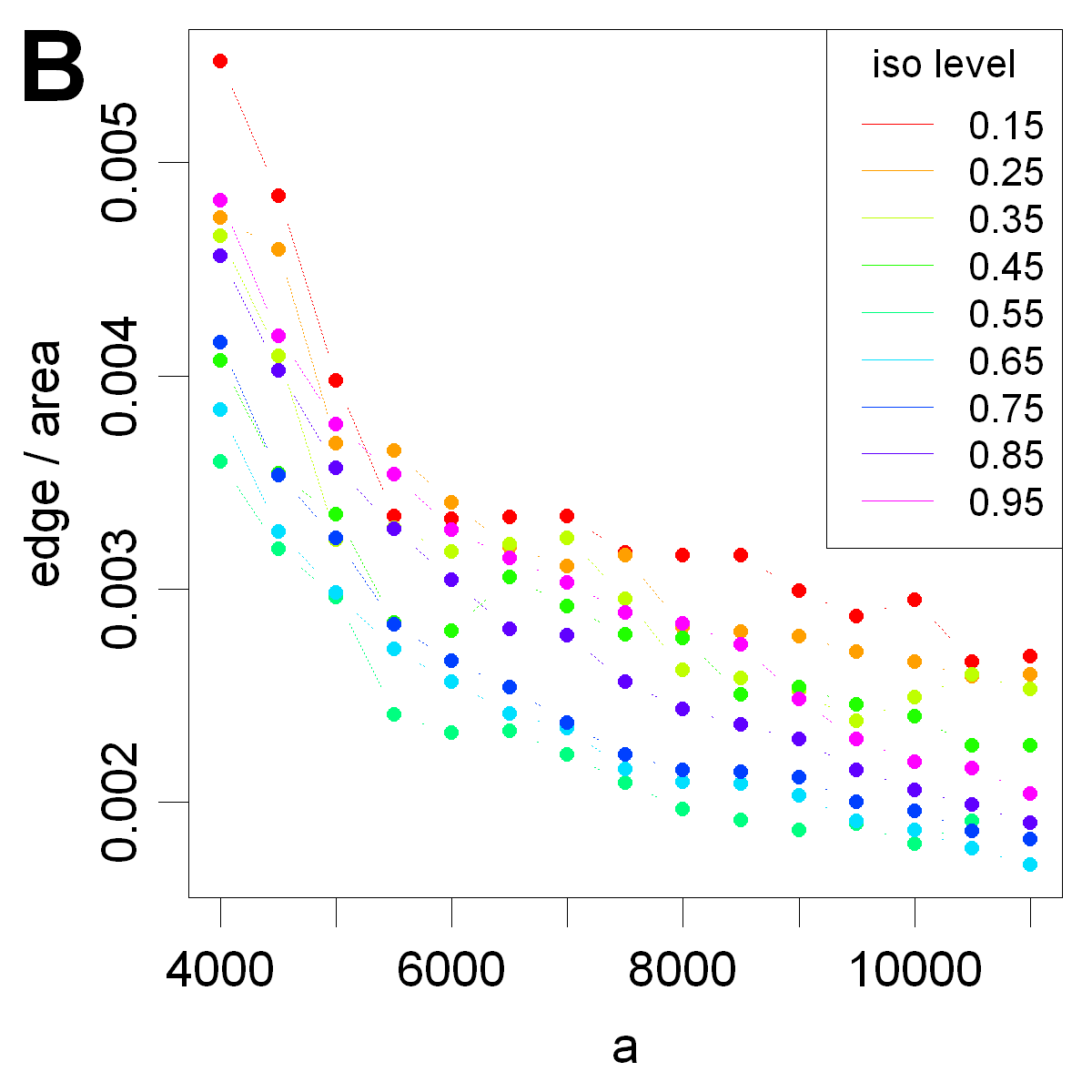


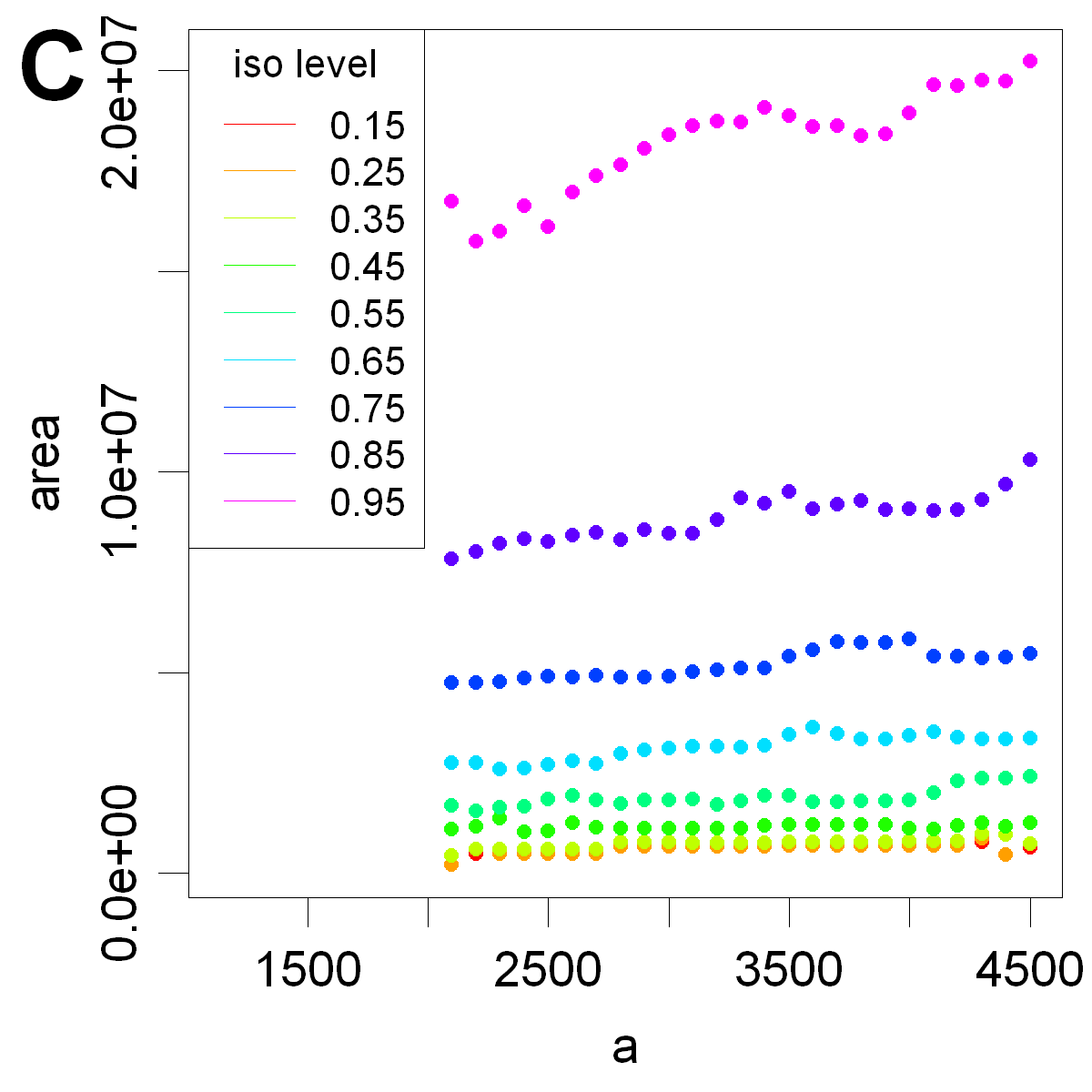

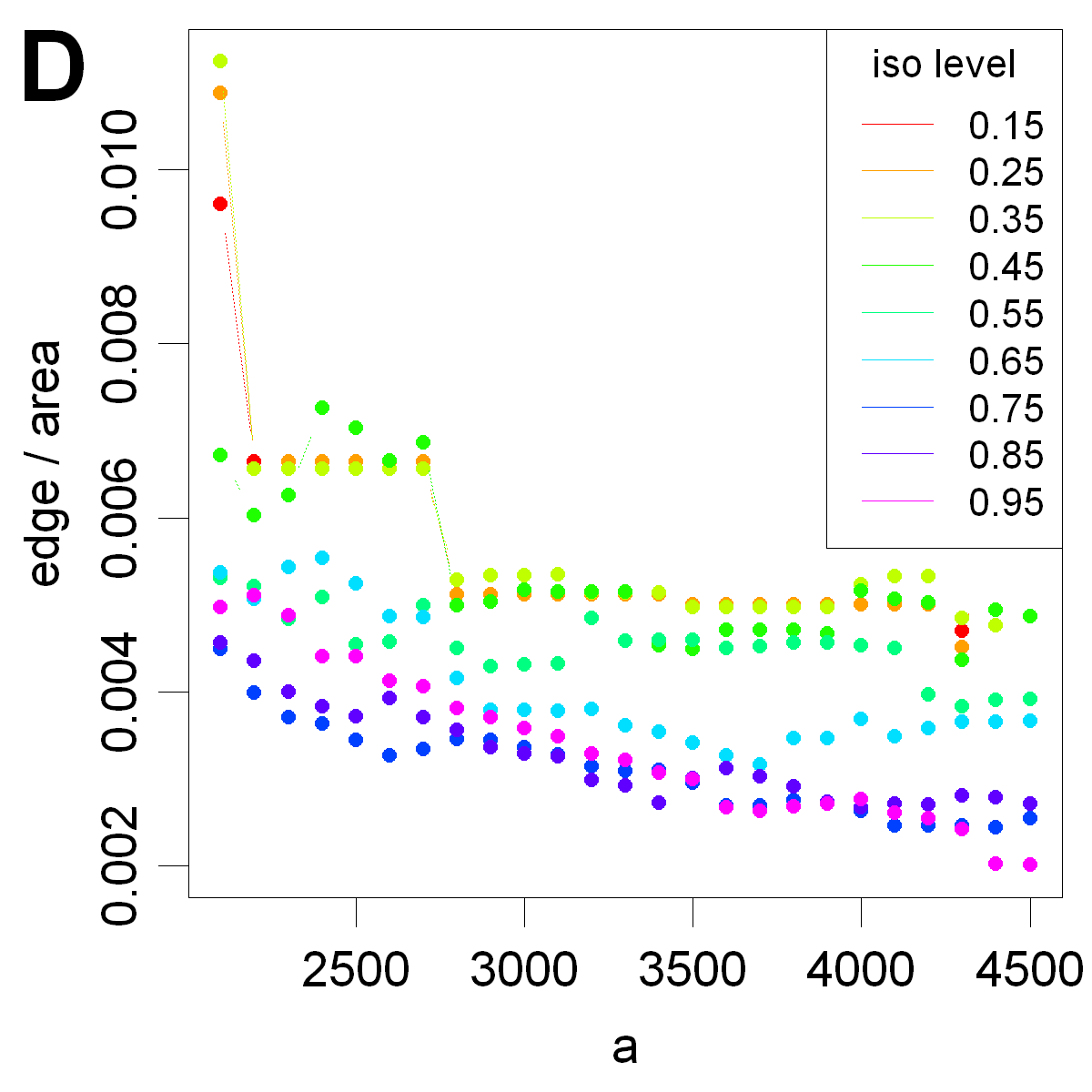


Figure S7

On left *a* vs. isopleth area curves for *s*=0.01 for the female (**A**) and male (**C**) springbok. On right *a* vs. isopleth edge:area ratio for female (**B**) and male (**D**).


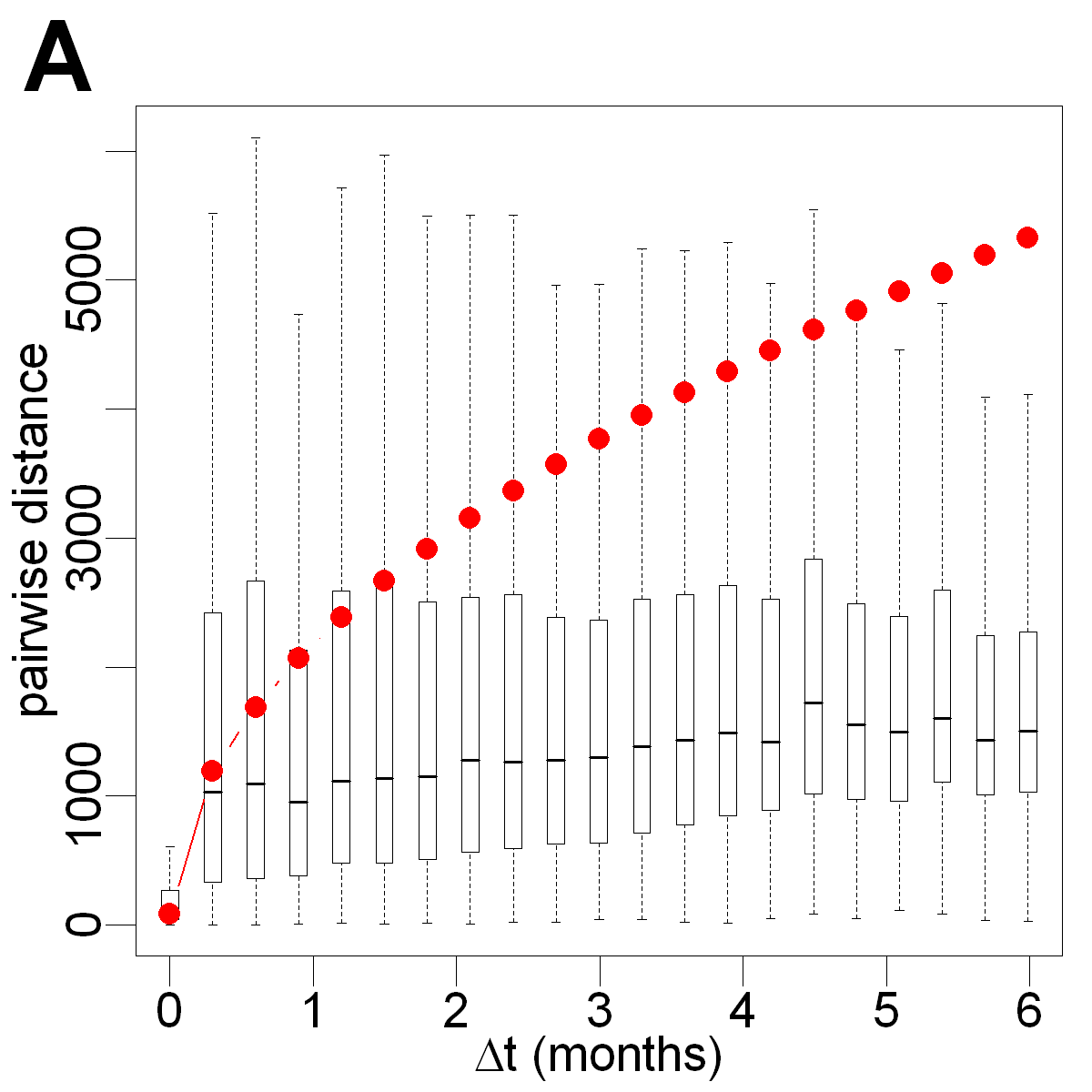

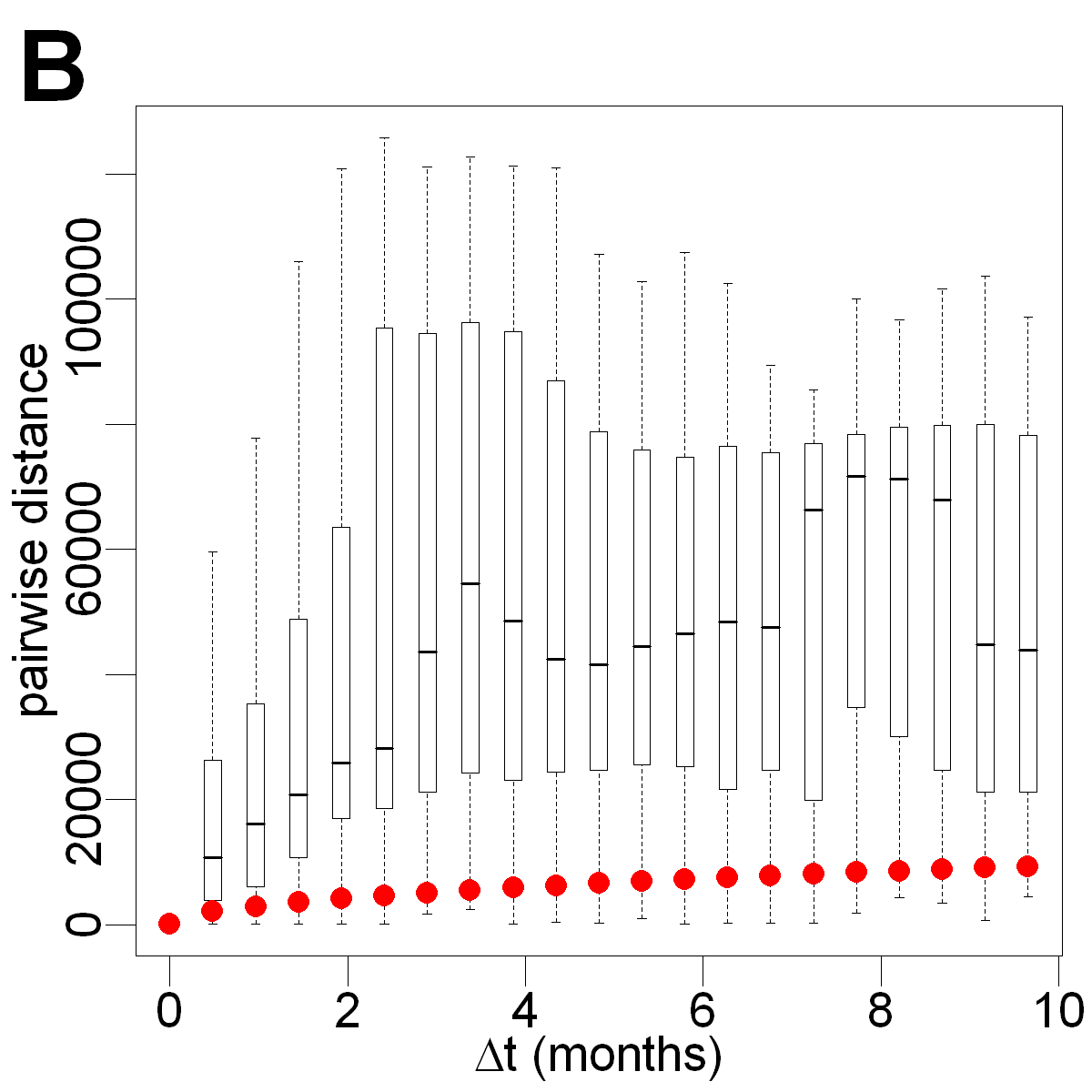


Figure S8.

Actual vs. predicted distance from a diffusion model of movement for the female (**A**) and territorial male (**B**) springbok. The red line is the predicted diffusion distance while the box and whisker plots are the distribution of actual distances for all pairs of points Δt apart on the x-axis.
